# Isolation of living dopaminergic neurons labeled with a fluorescent ligand of the dopamine transporter from mouse substantia nigra as a new tool for basic and applied research

**DOI:** 10.3389/fnmol.2022.1020070

**Published:** 2022-12-09

**Authors:** Dmitry Troshev, Victor Blokhin, Valeria Ukrainskaya, Anna Kolacheva, Michael Ugrumov

**Affiliations:** ^1^Laboratory of Neural and Neuroendocrine Regulations, Koltzov Institute of Developmental Biology of the Russian Academy of Sciences, Moscow, Russia; ^2^Laboratory of Biocatalysis, Shemyakin–Ovchinnikov Institute of Bioorganic Chemistry, Russian Academy of Sciences, Moscow, Russia

**Keywords:** dopaminergic neurons, substantia nigra, Parkinson’s disease, FACS, C57BL/6 mice, 1-methyl-4-phenyl-1,2,3,6-tetrahydropyridine, qPCR

## Abstract

Dopaminergic neurons (DNs) of the nigrostriatal system control the motor function, and their degeneration leads to the development of Parkinson’s disease (PD). A stumbling block in the study of DNs in the whole substantia nigra (SN) is the lack of tools to analyze the expression of most of the genes involved in neurotransmission, neurodegeneration, and neuroplasticity, since they are also expressed in other cells of the SN. Therefore, this study aimed to develop a fluorescence-activated cell sorting method for isolating living DNs from the SN of wild-type mice using two fluorescent dyes, DRAQ5 (nuclear stain) and a dopamine uptake inhibitor GBR 12909 coupled to a fluorophore (DN stain). We have developed a method for selecting a population of DNs from the SN of mice, as evidenced by: (i) immunopositivity of 95% of the sorted cells for tyrosine hydroxylase, the first enzyme of dopamine synthesis; (ii) the sorted cells expressing the genes for specific proteins of the dopaminergic phenotype, tyrosine hydroxylase, the dopamine transporter, and vesicular monoamine transporter 2 and non-specific proteins, such as aromatic L-amino acid decarboxylase, non-specific enzyme of dopamine synthesis. We then compared the changes in gene expression found in the sorted DNs and in the SN homogenate in a PD model we developed, reproduced in mice by treatment with 1-methyl-4-phenyl-1,2,3,6-tetrahydropyridine (MPTP). Using quantitative PCR, we obtained evidence of the same changes in the expression of specific genes in the sorted DNs of SN and in the SN homogenate of a MPTP mouse model of PD, compared with the control. The undoubted advantage of our approach is the possibility of obtaining a large amount of readily available and relatively cheap primary material (SN) from wild-type mice, which can be used to solve both research and applied problems. In addition, this method can be easily adapted to the isolation of DNs from the SN in other animal species, including non-human primates.

## Introduction

Nigrostriatal dopaminergic neurons (DNs) are crucial for the central regulation of motor function, and their death leads to the development of Parkinson’s disease (PD; Agid, 1991; Dauer and Przedborski, 2003). It is believed that the motor symptoms that serve to diagnose PD appear 20–30 years after the onset of the disease with a threshold degradation of the nigrostriatal dopaminergic system. This includes a 70–80% loss of dopamine in the striatum and a 50–60% loss of DNs in the substantia nigra (SN; Agid, 1991; Kalia and Lang, 2015; Postuma and Berg, 2016; Blesa et al., 2017). It is not surprising that with such a high degradation of the nigrostriatal system, symptomatic treatment of patients with dopamine agonists is of low efficiency (Ugrumov, 2020).

Based on the foregoing, the main trend in the fight against PD is the development of early (preclinical) diagnosis, long before the appearance of motor symptoms. Solving this problem would open up broad prospects for the development of neuroprotective treatment for PD, which would slow down the death of DNs and, therefore, prolong the period of asymptomatic development of the disease, or, in other words, the period of normal physical and social activity of patients (Simpkins and Jankovic, 2003; Biglan and Ravina, 2007; Salamon et al., 2020; Ugrumov, 2020). These developments are of particular importance due to the currently predicted rapid increase in PD incidence (Dorsey et al., 2007; Marras et al., 2018). Most studies of PD pathogenesis are carried out on animal and cell models of PD, since obtaining pathological material (post-mortem) from PD patients is very limited at the clinical stage and impossible at the preclinical stage (Ugrumov et al., 2011; Jagmag et al., 2016).

Given the key role of nigrostriatal DNs in the regulation of motor behavior and in the pathogenesis of PD, new knowledge about the molecular mechanisms of their functioning, degeneration, and plasticity is especially in demand. One of such relevant indicators is the expression of functionally important genes. Despite the fact that the SN contains predominantly DNs (Yung et al., 1991; Nair-Roberts et al., 2008), only three genes for DN proteins, tyrosine hydroxylase (TH), the dopamine transporter (DAT), and vesicular monoamine transporter 2 (VMAT2), can be evaluated in the SN homogenate (Dickerson et al., 2009; Kozina et al., 2014; Alieva et al., 2017; Mingazov et al., 2018). Evaluation of the expression of other genes in DNs, encoding aromatic L-amino acid decarboxylase (AADC), proteins of the vesicular cycle, proteasome system, etc., is not available in the whole SN, since these genes are also expressed in other nigral cells (Yung et al., 1991; Nair-Roberts et al., 2008; Alieva et al., 2017; Agarwal et al., 2020).

There are several approaches to the selective assessment of gene expression in DNs, which often require the use of transgenic animals (Bye et al., 2015; Liu et al., 2017; Papathanou et al., 2019; Chohan et al., 2020; Costa et al., 2021). Despite the ease of detecting DNs in transgenic animals, the use of these animals has some disadvantages, manifested either in non-selective expression of the label (Papathanou et al., 2019), or in significant differences in molecular mechanisms and behavior in transgenic animals compared with wild-type animals (Chohan et al., 2020; Costa et al., 2021). It follows that methods for isolating nigrostriatal DNs from wild-type animals are in great demand, since they can be used for transcriptomic analysis, avoiding some problems associated with the use of transgenic animals. In this context, the synthesis of a fluorescent dopamine transporter ligand that specifically stains DNs has provided new opportunities for isolating these neurons (Blokhin et al., 2022).

The aim of this study has been to develop a method for isolating DNs from the SN obtained from wild-type mice for the subsequent quantitative assessment of gene expression. The use of wild-type mice makes it possible to work in any vivarium and obtain an unlimited amount of readily available and relatively cheap primary material (SN) that can be used to solve both research and applied problems. The objectives of our study were to: (1) develop a method for isolating DNs from the SN; (2) to show that the isolated DNs can be used to quantify gene expression in various functional states of these neurons. It should be emphasized that the development of this method in mice could be easily adapted to work with any other animal species, including non-human primates.

## Materials and equipment

### Animals

– Male C57BL/6 mice [Laboratory animal farm Stolbovaya (SCBMT RAMS, Stolbovaya, Moscow reg., Russia)] aged 8–12 weeks weighing 20–24 g were used. The animals were kept at a temperature of 22 ± 1°C, with a 12-h day-night cycle and free access to food and water.

### Anesthesia

– Isoflurane (Baxter) 5AGG9621.

### Reagents

– 1-Methyl-4-phenyl-1,2,3,6-tetrahydropyridine (MPTP) hydrochloride (Sigma-Aldrich) M0896
  **Caution**: This substance is toxic, so avoid direct exposure or inhalation. Before manipulations with MPTP, see a technical review of MPTP utility and safety (Przedborski et al., 2001).
– Sodium chloride (Sigma-Aldrich) S9625
– Potassium chloride (Sigma-Aldrich) P4504
– Calcium chloride (Sigma-Aldrich) C4901
– Magnesium sulfate (Sigma-Aldrich) M7506
– Sodium bicarbonate (Sigma-Aldrich) S5761
– D-(+)-glucose (Sigma-Aldrich) G6152
– HEPES (Sigma-Aldrich) H3375
– L-Ascorbic acid (Sigma-Aldrich) A4544
– Sodium phosphate dibasic (Amresco) A611-0404-159
– Sodium phosphate monobasic (Amresco) 0571-1KG
– Sodium hydroxide solution (Sigma-Aldrich) 415,413
– Dulbecco’s Modified Eagle Medium (Gibco) 11,960–044
– Papain from papaya latex (Sigma-Aldrich) P4762
  **Caution**: This substance is toxic, so avoid direct exposure or inhalation.
– Fetal bovine serum (Gibco) A3160801
– Hank’s Balanced Salt Solution (HBSS; Gibco) 14,025–092
– DRAQ5 (Abcam) ab108410
  **Caution**: This substance is toxic, so avoid direct exposure.
– Propidium iodide (PI; Sigma-Aldrich) P4170
  **Caution**: This substance is toxic, so avoid direct exposure.
– GBR 12909 dihydrochloride (Sigma-Aldrich) D052
– Dimethyl sulfoxide (Sigma-Aldrich) D8418
– RiboLock RNase inhibitor (Thermo Fisher Scientific) EO0381
– TRI Reagent (Sigma-Aldrich) 93,289
  **Caution**: This substance is toxic, so avoid direct exposure or inhalation.
– Paraformaldehyde (Sigma-Aldrich) 158,127
  **Caution**: This substance is toxic, so avoid direct exposure or inhalation.
– Gelatin from bovine skin (Sigma-Aldrich) G9382
– Cromium (III) potassium sulfate dodecahydrate (Sigma-Aldrich) 243,361
– Sodium azide (Sigma-Aldrich) S2002
  **Caution**: This substance is toxic, so avoid direct exposure or inhalation.
– Bovine serum albumin (Sigma-Aldrich) A7906
– Triton X-100 (Sigma-Aldrich) T9284
– Mounting medium with 4′,6-diamidino-2-phenylindole (DAPI) - Aqueous, Fluoroshield (Abcam) ab104139
– 1-Bromo-3-chloropropane (Sigma-Aldrich) B9673
– Isopropyl alcohol (Sigma-Aldrich) I9030
– Glycogen, RNA grade (Thermo Fisher Scientific) R0551
– Ethanol 95% (Acros Organics) 64–17-5
– PCR grade water (Evrogen) PB207S
– DNase I, RNase-free (Thermo Fisher Scientific) EN0521
– Maxima H Minus First Strand cDNA Synthesis Kit (Thermo Fisher Scientific) K1682
– qPCRmix-HS SYBR+LowROX (Evrogen) #PK156L
– Agarose (Helicon) Am-N605-0.05
– Ethidium bromide (Thermo Fisher Scientific) 15,585–011
– GeneRuller 100 bp DNA Ladder (Thermo Fisher Scientific) SM0241
– Oligonucleotide primers (Evrogen; Table 1)
– Sheep anti-TH antibody (Chemicon) AB1542
– Donkey anti-sheep gamma globulins conjugated to Alexa Fluor 488 (Invitrogen) A11015.

**Table 1:** Oligonucleotide primers used for qPCR.

**Gene**	**Forward primer**	**Reverse primer**	**PCR product length, bp**
CYC1 gene	5’-GCGGCCAGGGAAGTTGT-3’	5’-GCCAGTGAGCAGGGAAAATAC-3’	154
TH gene	5′- TCAGAGGAGCCCGAGGTC-3’	5’-GGGCGCTGGATACGAGAG-3’	152
DAT gene	5′- CATCAACCCACCGCAGAC -3’	5’-GAAGGCACCTCCACCATTT-3’	154
VMAT2 gene	5’-ATTGGCTTTCCTTGGCTCAT-3’	5’-GGTACGGCTGGACATTATTCTG-3’	172
AADC gene	5’-TCCCCACGGCTAGCTCATACCC-3’	5’-TTCCCCAGCCAGTCCATCATCA-3’	133
GFAP gene	5′-CATGCCACGCTTCTCCTTGT-3’	5′-ATCATCTCTGCACGCTCGCT-3’	123

### Reagent setup

#### MPTP solution – Timing: 15 min

To model PD in mice, MPTP was used at a dose of 12 μg/g animal. Since we are using MPTP hydrochloride (209.72 g/mol) and not MPTP (173.25 g/mol), the amount of drug administered to animals is calculated аs follows: molar mass of MPTP hydrochloride/molar mass of MPTP x preliminary selected dose of MPTP. In our case: 209.72/173.25 × 12 = 14.526 μg/g. MPTP hydrochloride was diluted in saline at the rate of 5 μl of saline per 1 g of mouse weight. That is, MPTP hydrochloride concentration = 14.526/5 = 2.905212 μg/μl. For example, a 20 g animal would be injected with 100 μl of MPTP hydrochloride solution at a concentration of 2.90521 μg/μl or 290.521 μg of the drug per 20 g, which corresponds to a preliminary selected dose of MPTP hydrochloride (14.526 μg/g) or MPTP (12 μg/g).

#### Krebs-ringer solution (500 ml) – Timing: 20 min

To 450 ml milli-Q water successively add: 3.5064 g sodium chloride (120 mM), 0.1789 g potassium chloride (4.8 mM), 0.111 g calcium chloride (2.0 mM), 0.0782 g magnesium sulfate (1.3 mM), 1.0501 g sodium bicarbonate (25 mM), 0.9008 g D-(+)-glucose (10.1 mM), 2.383 g HEPES (20 mM), and 0.0088 g L-ascorbic acid (0.13 mM). The solution is constantly stirred. Raise the volume of the solution to 500 ml, and adjust the pH to 7.2–7.4.

#### Papain solution (500 μl) – Timing: 40 min

To 1 mg of papain from papaya latex, add 500 μl of Dulbecco’s Modified Eagle’s Medium. Papain is then activated by incubation for 30 min at 37°C. Papain solution prepare *ex tempore*.

#### 10 μg/Ml PI solution (500 μl) – Timing: 10 min

Prepare PI stock solution (1 mg/ml): add 1 mg PI to 1 ml milli-Q water, stir until PI is completely dissolved. The stock solution is stored at 4°C protected from light for up to 6 months. To obtain 500 μl 10 μg/ml PI solution add 5 μl PI to 500 μl HBSS and stir.

#### 10 μM GBR 12909 solution (1 ml) – Timing: 20 min

Prepare stock solution of GBR 12909 (1 mM): add 300 μl of dimethyl sulfoxide to 5.23 mg GBR 12909, stir. Add 700 μl milli-Q water to the resulting solution, stir until GBR 12909 is completely dissolved. To make 10 μM GBR 12909 solution, add 990 μl HBSS to 10 μl GBR 12909 stock solution. GBR 12909 solution prepare *ex tempore*.

#### 0.2 M phosphate buffer (500 ml) – Timing: 30 min

In 450 ml milli-Q water dissolve 2.3 g sodium phosphate monobasic and 11.47 g sodium phosphate dibasic. Raise the volume of the solution to 500 ml and adjust the pH to 7.2–7.4.

#### 0.02 M phosphate buffer saline (100 ml) – Timing: 10 min

Mix 90 ml milli-Q water and 10 ml 0.2 M phosphate buffer. Add 0.9 g of sodium chloride to the resulting solution. Adjust the pH of the solution to 7.2–7.4.

#### Fixative solution (4% paraformaldehyde; 200 ml) – Timing: 90 min

Add 8 g paraformaldehyde to 100 ml milli-Q water and dissolve it at 60–65°C with constant stirring. After paraformaldehyde dissolves, add 1–2 drops of sodium hydroxide solution to clear the solution. The solution is then cooled and filtered. To obtain a 4% paraformaldehyde solution, add 100 ml 0.2 M phosphate buffer to the resulting solution.

### Equipment

– Centrifuge tube (0.2 ml; SSIbio) 3,225–00
– Centrifuge tube (1.5 ml; SSIbio) 1,260–00
– Pipette tips: 10 μl/200 μl/1000 μl (OMNITIP) 81,710/83710/85710
– Pipette tips for mechanical tissue dissociation (Corning) 4,864
– 40 μm Cell Strainer (Falcon) 352,340
– Sorting Chip-100 μm (Sony) LE-C3210
– Glass slides (Thermo Fisher Scientific) AAAA000001##12E
– Cover slips (Corning) 2,975–225
– Mechanical pipettes: 0.2–2.5 μl/2–20 μl/20–200 μl/100–1,000 μl (Eppendorf)
– Vortex (ELMI)
– Milli-Q lab water system (Millipore)
– Vibratome: Microm HM 650 V (Thermo Fisher Scientific)
– Dissecting microscope: Leica M60 (Leica Biosystems)
– Centrifuge: 5424 R (Eppendorf)
– Incubator: MIR–153 (SANYO)
– Multirotation shaker: Multi Bio RS-24 (BioSan)
– Flow cytometer: CytoFLEX S (Beckman Coulter)
– Cell sorter: Sony SH800S (Sony)
– ImageStreamx mk II system (Luminex Corp.)
– Fluorescent microscope: Zeiss Axio Observer equipped with a Zeiss Colibri LED illumination system and a Zeiss AxioCam MRc camera (Zeiss)
– NanoDrop 8,000 (Thermo Fisher Scientific)
– QuantStudio 12 k Flex (Applied Biosystems)
– PowerPac^™^ Basic Power Supply (Bio-Rad Laboratories)
– Horizontal electrophoresis system SE-1 (Helicon)
– ChemiDoc Touch (Bio-Rad Laboratories).

### Equipment setup

#### Gelatin-coated slides – Timing: Several hours

Put the glass slides in a rack and dip first in milli-Q water and then in 95% ethanol. Transfer the rack with glasses to an oven and dry them for 1–1.5 h at 40–45°C. To 300 ml unboiled milli-Q water add 3 g gelatin from bovine skin, 0.3 g cromium (III) potassium sulfate dodecahydrate and 0.06 g sodium azide. Dissolve the gelatin in a fume hood with constant stirring. Filter the gelatin solution and cool to 40–50°C. Pour the gelatin solution into the bath and immerse the rack with the dried slides in this solution. Then the bath with slides is placed in an oven and dried for at least 2 h at 40–50°C until the gelatin is completely solidified. Store slides at 4°C.

### Software

– CytExpert software (Beckman Coulter)
– SH800 Software (Sony)
– IDEAS software (Luminex Corp.)
– AxioVision 40 V 4.8.2.0 software (Zeiss)
– ImageJ software^1^
– GraphPad Prism 6 software (GraphPad Software).

## Materials and methods

### Animals and experimental procedures

**Figure 1:**
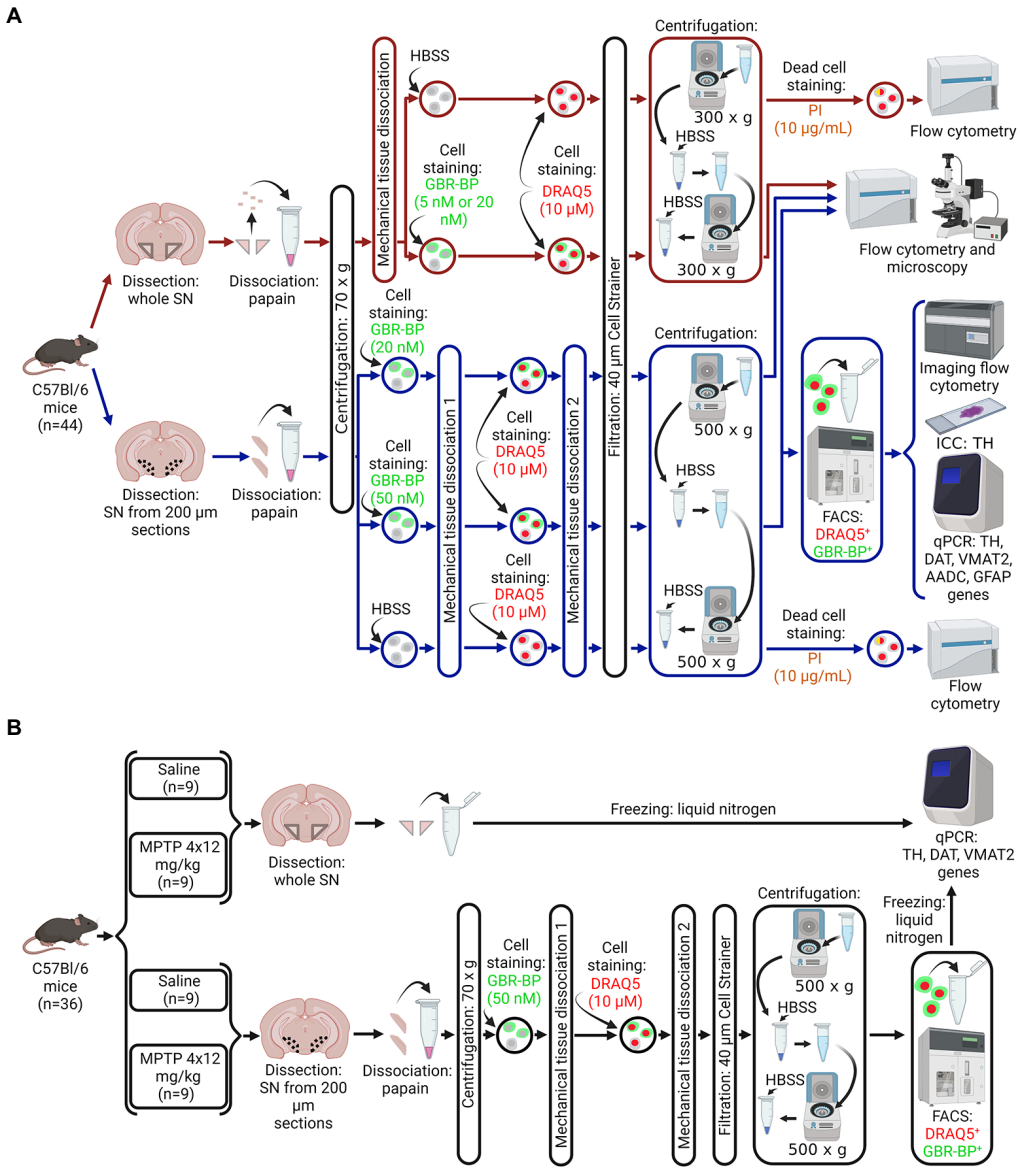
Design of experiments. **(A)** Protocol for isolating a population of dopaminergic neurons (DNs) from a cell suspension of the whole substantia nigra (SN) or vibratome sections of the SN obtained from intact mice for subsequent assay by flow cytometry, immunocytochemistry, and quantitative polymerase chain reaction (qPCR). **(B)** Sample preparation for the analysis of gene expression of DN marker proteins in a homogenate of a whole SN or in a population of DNs isolated from a cell suspension of SN vibratome sections obtained from mice in a model of the early clinical stage of Parkinson’s disease using 1-methyl-4-phenyl-1,2,3,6-tetrahydropyridine (MPTP) and control (saline). Created with BioRender.com. AADC, aromatic L-amino acid decarboxylase; DAT, dopamine transporter; DRAQ5, a nuclear dye, FACS, fluorescence-activated cell sorting; GBR-BP, a specific DN dye; GFAP, glial fibrillary acid protein; HBSS, Hank’s balanced salt solution; PI, propidium iodide; TH, tyrosine hydroxylase; VMAT2, vesicular monoamine transporter 2.

C57BL/6 mice (*n* = 80) were divided into three groups (Figure 1). The first group (*n* = 44) included intact mice (Figure 1A). The second group included mice that were subcutaneously injected with MPTP 4 times at a single dose of 12 mg/kg with an interval of 2 h between injections (*n* = 18). The third group included mice (*n* = 18) that were subcutaneously injected with 0.9% sodium chloride (saline) 4 times with an interval of 2 h between injections. Mice of the second and third groups were kept for 2 weeks under normal laboratory conditions after injections until the material for quantitative polymerase chain reaction (qPCR) and immunohistochemistry was obtained (Figure 1B).

### Obtaining the substantia nigra – Timing: 5 min for obtaining the whole substantia nigra, 12 min for obtaining the substantia nigra from vibratome sections

Mice belonging to the three groups (see above) were decapitated under 2.4% isoflurane anesthesia. After that, the brain was removed and, in some cases, the whole SN was excised as described earlier (Ugrumov et al., 2011; Kozina et al., 2014). In other cases, using a vibratome, we prepared a series of frontal 200 μm sections of the brain with the SN along the rostro-caudal direction from −2.54 to −3.88 mm of the bregma according to the mouse brain atlas (Paxinos and Franklin, 2001). The vibratome sections were prepared in ice-cold Krebs-Ringer solution. The SN was cut out from the vibratome frontal sections of the brain with a razor blade under a dissecting microscope. Although we tried to carefully excise the compact part of the SN, a small amount of border tissue may have been included in the final sample. The SN obtained as a whole piece or in vibratome sections from all mice of the first group (*n* = 44), as well as from half of the mice of the second (*n* = 9) and third (*n* = 9) groups, was used to prepare a cell suspension for subsequent assay and sorting of DNs. The whole SN obtained from the second half of the mice of the second (*n* = 9) and third (*n* = 9) groups was frozen and stored at −70°C until qPCR was performed (Figure 1B).

### Preparation of a cell suspension from the whole substantia nigra and its processing – Timing: 70 min

DNase and RNase free equipment was used for preparing a cell suspension and its further processing. In the first step of cell suspension preparation, the brain’s whole SN was cut with a razor blade into small pieces. These were transferred to a 1.5 ml centrifuge tube, containing 100 μl cooled (4°C) Dulbecco’s modified eagle medium. The tubes with the small pieces of SN were centrifuged for 30 s at 70× g and 20°C. The supernatant was collected and 50 μl of papain solution was added. **Critical Step**: the precipitate was shaken on a vortex and incubated in the same solution for 30 min at 37°C with stirring, which promotes the dissociation of the nervous tissue (Martin et al., 2017; Turko et al., 2019). The enzymatic action of papain was stopped by adding cooled (4°C) fetal bovine serum into the tubes at a final concentration of 10% (v/v). The resulting solution was stirred on a vortex. Thereafter, neural tissue was precipitated by centrifugation at 70× g for 30 s and 4°C and then washed twice in cold 100 μl HBSS. The tissue was then re-centrifuged at 70× g for 30 s and 4°C. The precipitate was resuspended in 50 μl of HBSS and pumped 30–40 times with a 200 μl mechanical pipette through a tip with a 0.5 mm diameter until visual dissociation of the tissue pieces (**Critical Step**: avoid the formation of air bubbles during mechanical dissociation).

DNs contained in the cell suspension of the SN were stained with two fluorescent dyes. One dye, DRAQ5, stains the nuclei of both living and fixed cells (Smith et al., 2004). Another dye, GBR-BODIPY (GBR-BP), specifically stains DNs by binding to DAT and subsequent internalization into the neurons (Blokhin et al., 2022). These dyes have non-overlapping emission spectra: DRAQ5 with a maximum at 697 nm; GBR-BP with a maximum at 511 nm. It should be noted that the nuclear dye (DRAQ5) with cytotoxic properties might cause cell death during their long-term cultivation, which is a limitation of our method for sorting DNs. The SN cell suspension was incubated in 50 μl HBSS containing 5 nM or 20 nM GBR-BP in the dark at 37°C for 15 min with stirring (Figure 1A). Then, 10 μM DRAQ5 was added and the cell suspension was incubated for another 15 min under the same conditions. The stained cell suspension was passed through a moistened 40 μm cell strainer. The cells were then precipitated by centrifugation at 300× g for 5 min at 4°C, washed in 100 μl HBSS, and re-centrifuged under the same conditions. The precipitate was resuspended in 50 μl HBSS and the suspension of living cells was stored in the dark at 4°C until fluorescent microscopy or flow cytometry.

To assess cell viability, the whole SN cell suspension incubated before with 10 μM DRAQ5 was precipitated by centrifugation at 300× g for 5 min at 4°C, resuspended in 50 μl PI solution and incubated for 5 min at 4°C with shaking. The resulting cell suspension was stored in the dark at 4°C until analysis by flow cytometry. The cell viability was expressed as a ratio of the number of dead cells stained with PI to the total number of cells stained with DRAQ5.

The GBR-BP staining specificity of the whole SN cell suspension was tested by incubating it first with 10 μM GBR 12909, a DAT inhibitor, for 10 min at 37°C, and then with 10 μM GBR 12909 and 5 nM or 20 nM GBR-BP for 30 min at 37°C. The cells were then precipitated by centrifugation at 300× g for 5 min at 4°C, resuspended in 50 μl HBSS and stored in the dark at 4°C until fluorescent microscopy. This control was used in a previous study to assess the specificity of GBR-BP staining of DNs derived from primary culture of mouse embryonic mesencephalon and LUHMES cells (Blokhin et al., 2022).

### Preparation of a cell suspension from vibratome sections of the substantia nigra and its assay – Timing: 70–75 min

The brain’s SNs excised from frontal vibratome sections (hereinafter referred to as SN vibratome sections) were transferred to microcentrifuge tube containing 100 μl of cooled (4°C) Dulbecco’s modified eagle medium. As in the preparation of a cell suspension from the whole SN, SNs excised from one animal was used to obtain one cell suspension. It should be noted that all solutions that were further used for fluorescence-activated cell sorting (FACS) contained 100 U/ml of the RNase inhibitor RiboLock. As for obtaining a cell suspension from the whole SN, tubes with SN vibratome sections were centrifuged at 70× g for 30 s at 20°C. The precipitate was incubated with papain solution and centrifuged again at 70× g for 30 s at 4°C.

For specific staining of DNs, the precipitate was incubated for 15 min in 50 μl HBSS containing 20 nM or 50 nM GBR-BP (Figure 1A), followed by partial cell dissociation by pipetting 15 times through a tip with a 0.5 mm diameter (Critical Step: avoid the formation of air bubbles during mechanical dissociation). This cell suspension, still containing cell conglomerates, was incubated with 10 μM DRAQ5 and 20 nM or 50 nM GBR-BP for 15 min at 37°C and dissociated again by pipetting until the complete disappearance of cell conglomerates (Critical Step: avoid the formation of air bubbles during mechanical dissociation). The stained cell suspension was passed through a moistened 40 μm cell strainer. The cells were then centrifuged at 500× g for 5 min at 4°C, washed in 100 μl HBSS, and centrifuged again under the same conditions. Thereafter, the supernatant was collected and the precipitate was resuspended in 50 μl HBSS. This cell suspension was stored in the dark at 4°C until fluorescence microscopy, flow cytometry, or FACS.

To assess cell viability, the SN vibratome sections suspension incubated before with 10 μM DRAQ5 was precipitated by centrifugation at 500× g for 5 min at 4°C and resuspended in 50 μl PI solution and incubated for 5 min at 4°C with shaking. The resulting suspension was stored in the dark at 4°C until analysis by flow cytometry.

The GBR-BP staining specificity of SN vibratome sections was assessed after inactivating of papain and washing in HBSS by incubation first with 10 μM GBR 12909 for 10 min at 37°C and then with 10 μM GBR 12909 and 20 nM or 50 nM GBR-BP in accordance with the protocol of specific staining of DNs from whole SN (see above). The cells were then precipitated by centrifugation at 500× g for 5 min at 4°C, resuspended in 50 μl HBSS and stored in the dark at 4°C until fluorescent microscopy.

### Flow cytometry and fluorescence-activated cell sorting

Before flow cytometry/FACS, the volume of the analyzed suspensions was adjusted to 500 μl with cold HBSS (4°C). Cell suspensions obtained from the whole SN or SN vibratome sections were assessed in a flow cytometer or a cell sorter using a sorting chip with a diameter of 100 μm at a pressure of 21–23 psi. The threshold staining levels for DRAQ5, GBR-BP, and PI were determined by analysing the unstained cell suspension and single stain controls for dyes with fluorescence spectra overlap: DRAQ5 and PI (Supplementary Figures 1, 2). DRAQ5 was excited with a 638 nm laser and detected using a 712/25 nm (for flow cytometer) or 720/60 nm (for cell sorter) band pass filter. GBR-BP was excited with a 488 nm laser and detected with a 525/40 nm (for flow cytometer) or 525/50 nm (for cell sorter) band pass filter. PI was excited with a 488 nm laser and detected with a 574/26 nm (for flow cytometer) band pass filter. The forward scatter and side scatter profiles of the unstained cell suspension were determined to outline the population containing cells – cell population. For each cell suspension, 200,000 events (all detected particles) were analysed by flow cytometry. FACS was used only for the assay of the cell suspensions obtained from SN vibratome sections stained with 10 μM DRAQ5 and 50 nM GBR-BP. All cells obtained from SN vibratome sections suspension stained with a fluorescence intensity above a predetermined threshold (DRAQ5^+^/GBR-BP^+^) were harvested into a centrifuge tube. The tubes with sorted cells were stored at 4°C before analysis by imaging flow cytometry and immunocytochemistry. Cells for qPCR were sorted into a centrifuge tubes containing 50 μl TRI Reagent and 100 U/ml RiboLock. 250 μl TRI Reagent was then added to these tubes and after mixing, they were frozen in liquid nitrogen and stored at −70°C until total RNA extraction. Histograms were plotted and the results were processed using CytExpert software and SH800 software.

### Imaging flow cytometry

For imaging flow cytometry, images of dissociated SN cells stained with DRAQ5 and GBR-BP were obtained, first before sorting and then after sorting, using the ImageStreamx mk II system equipped with 488 nm and 642 nm lasers, at 40x objective magnification. Image analysis was performed using IDEAS software. First, images in focus were selected using the gradient root mean square scores histogram, and then the area containing the cells was outlined using image area values from 100 to 225 pixels and aspect ratio intensity from 0.5 to 1. These settings allow single cells to be counted, but not cell conglomerates. Further outlining selection of the analyzed cell populations was performed by the intensity of DRAQ5 and GBR-BP fluorescence. DRAQ5 and GBR-BP fluorescent intensity threshold levels were set according to the same protocol as for flow cytometry/FACS of neurons (see above; Supplementary Figure 3).

### Immunocytochemistry – Timing: 15 h

The cells of the suspension obtained from SN vibratome sections were immunostained for TH before sorting (*n* = 3) and after sorting (*n* = 3). For this, the cell suspension was centrifuged at 2000× g for 5 min at 4°C. The supernatant was removed and the precipitate was resuspended in 200 μl of 4% paraformaldehyde and incubated for 10 min at 4°C. The fixed cell suspension was then centrifuged at 2,000× g for 5 min at 4°C. The precipitate was washed twice with 200 μl of phosphate buffer saline and centrifuged again at 2,000× g for 5 min at 4°C. Then, 100 μl of phosphate buffer saline was added to the precipitate, which was resuspended. The resulting cell suspension was transferred to a gelatin-coated slide and dried at 37°C.

The suspension put on the slide was incubated successively in phosphate buffer saline: (a) with 3% bovine serum albumin and 0.1% Triton X-100 for 30 min at 20°C; (b) with sheep anti-TH antibody (1:500), 1% bovine serum albumin, and 0.02% Triton X-100 overnight at 4°C; (c) with donkey anti-sheep gamma globulins conjugated to Alexa Fluor 488 (1:700) for 2 h at 20°C. After being washed in phosphate buffer saline, the resulting preparations were embedded in a medium with DAPI.

### Microscopy

Microscopy of cell suspension first stained with GBR-BP and then fixed and immunostained for TH, was performed using a fluorescent microscope. Images were obtained at objective magnifications of 20×, 40×, and 63× and analysed using AxioVision 40 V 4.8.2.0 software. On each slide, the proportion of TH-immunopositive cells (in %) was determined, in relation to all cells in 100 fields of view (each = 0.033 mm^2^), taken as 100%. The total number of cells was determined by the number of DAPI-stained nuclei.

The ImageJ software was used to assess the fluorescence intensity of a cell suspension obtained from whole SN and stained with 5 nM and 20 nM GBR-BP, as well as a cell suspension obtained from vibratome sections of SN, preliminarily stained with 20 nM or 50 nM GBR-BP (suspensions were placed between two cover glasses). The specificity of GBR-BP cell staining was assessed as proportion of stained cells among all the cells detected using differential interference contrast microscopy in cell suspensions. In each case, 10 randomly selected fields of view (each = 0.138 mm^2^) were analyzed.

The images were converted to an 8-bit format and the “Area,” “Integrated Density,” and “Mean Gray Value” of the cells were measured. Once these parameters were obtained, the corrected total cell fluorescence (CTCF) of GBR-BP-stained cells was calculated as follows:

CTCF = “Integrated Density” − (“Area of selected cells” ∙ “Mean fluorescence of background values”).

### RNA extraction and quantitative polymerase chain reaction

Extraction of the total RNA from sorted cells was performed by adding 700 μl TRI Reagent to 300 μl of thawed TRI Reagent containing sorted cells obtained from some animals of the 1st group (*n* = 6), half of the animals (*n* = 9) of the 2nd group (MPTP administration), and half of the animals (*n* = 9) of the 3rd group (saline administration). Then, 100 μl 1-bromo-3-chloropropane was added and the incubation of the solutions was continued at 20°C for 15 min, vortexing every 3 min. Phases were separated by centrifugation at 21,000× g for 15 min at 4°C. The aqueous phase containing RNA was transferred to a new 1.5 ml centrifuge tube and 500 μl isopropyl alcohol was added. For better RNA precipitation, 1 μl glycogen was added. The sample was vortexed and incubated for 10 min at 20°C, after which RNA was precipitated by centrifugation at 21,000× g for 10 min at 4°C. The supernatant was removed and the precipitate was washed three times in 1 ml 80% ethanol and centrifuged at 21,000× g for 10 min at 4°C. After the last centrifugation, ethanol was removed and the RNA precipitate was air-dried for 15 min. Then RNA was dissolved in 20 μl PCR grade water.

In addition to the SN cell suspension, whole SNs obtained from half of the animals (*n* = 9) of the 2nd group (MPTP administration) and half of the animals (*n* = 9) of the 3rd group (saline administration) were used to extract total RNA for qPCR. 500 μl TRI Reagent was added to the obtained samples. This mixture was incubated for 5 min at 20°C and homogenized by pipetting, and after that, another 500 μl TRI Reagent was added. Extraction of the total RNA was then performed in the same way as extraction of the total RNA was carried out from sorted cells (see the paragraph above).

The RNA concentration in all samples was measured using a NanoDrop 8,000. Residual genomic DNA was removed using DNase I, RNase-free according to the manufacturer’s protocol. Expression of the key DN genes for TH, DAT, VMAT2, and AADC, as well as the astrocyte gene for glial fibrillary acidic protein was assessed in sorted cells and in whole SN by qPCR. The cytochrome C1 gene was used as a reference gene. Complementary DNA was synthesized from 150 ng total RNA using the Maxima H Minus First Strand cDNA Synthesis Kit according to the manufacturer’s protocol. Complementary DNA concentration was measured using a NanoDrop 8,000. qPCR was performed using qPCRmix-HS SYBR+LowROX on a QuantStudio 12 k Flex DNA amplifier. Oligonucleotide primers were used to determine the expression of key DN markers and glial fibrillary acid protein (Table 1).

For qPCR, 500 ng complementary DNA was used. Amplification was carried out according to the following protocol: hold stage – at 50°C for 2 min and then at 95°C for 10 min; PCR stage – at 95°C for 15 s and then at 60°C for 1 min (50 cycles); melt curve stage – sequentially at 95°C for 15 s, at 60°C for 1 min, and at 95°C for 15 s. After the end of the reaction, the amplified products were separated at a voltage of 100 V in 1.5% agarose gel containing ethidium bromide. DNA fragments were detected using ChemiDoc Touch.

A comparative analysis of TH,DAT, VMAT2, and AADC gene expression in the sorted DNs and in the whole SN of mice treated with MPTP or saline was carried out by qPCR, in the same way as when evaluating the gene expression in the sorted DNs of intact animals (see above). In this study, primers for TH, DAT, VMAT2, AADC, and cytochrome C1 genes were used (Table 1). Changes in TH, DAT, VMAT2, and AADC gene expression were calculated using the 2^−ΔΔC(t)^ method.

### Statistics

Statistical analysis was performed using GraphPad Prism 6 software. Groups were compared for normality by using the D’Agostino & Pearson test. For pairwise comparison, we used the unpaired t-test or the Mann–Whitney test, depending on the type of distribution. For the analysis of 3 or more groups, we used the one-way ANOVA test, followed by Tukey’s *post-hoc* test for multiple comparisons. The results are presented as mean ± SEM. Differences were considered significant at *p* < 0.05.

## Results

### Development of a protocol for obtaining a population of GBR-BODIPY-stained dopaminergic neurons from the substantia nigra of intact mice

At the initial stage of this study, we developed an optimal protocol for SN cell dissociation, based on previous experience in cell dissociation of the nervous tissue of other areas of the brain (Guez-Barber et al., 2011; Turko et al., 2019). For this, SN excised from the brain was first cut mechanically, and then the extracellular matrix was partly degraded using papain. Due to the much smaller volume of the SN compared to those areas of the brain that were dissociated in previous studies (Guez-Barber et al., 2011; Turko et al., 2019), SN was cut into small pieces which were then incubated with papain in centrifuge tubes in a small volume of solution (50 μl). To ensure a uniform enzymatic effect on the tissue, the papain solution with SN in tubes was constantly stirred, as was done in other similar studies during the dissociation of nervous tissue (Xu et al., 2012; Miyazaki et al., 2020).

After the dissociation of SN, which is heterogeneous in cellular composition, it was necessary to identify DNs in the resulting cell suspension. For this, along with DRAQ5, which stains cell nuclei, GBR-BP, a recently manufactured specific fluorescent dye for monoaminergic neurons, was used (Lavrova et al., 2019; Blokhin et al., 2022). Indeed, GBR-BP has been shown to specifically stain DNs in a primary culture of mouse embryonic mesencephalon cells and LUHMES cells (Blokhin et al., 2022).

At the first stage of developing the optimal protocol for specifically staining DNs in the SN cell suspension, GBR-BP was used at a concentration of 5 nM, which was previously used to stain DNs in a primary culture of mouse mesencephalon (Blokhin et al., 2022).

**Figure 2:**
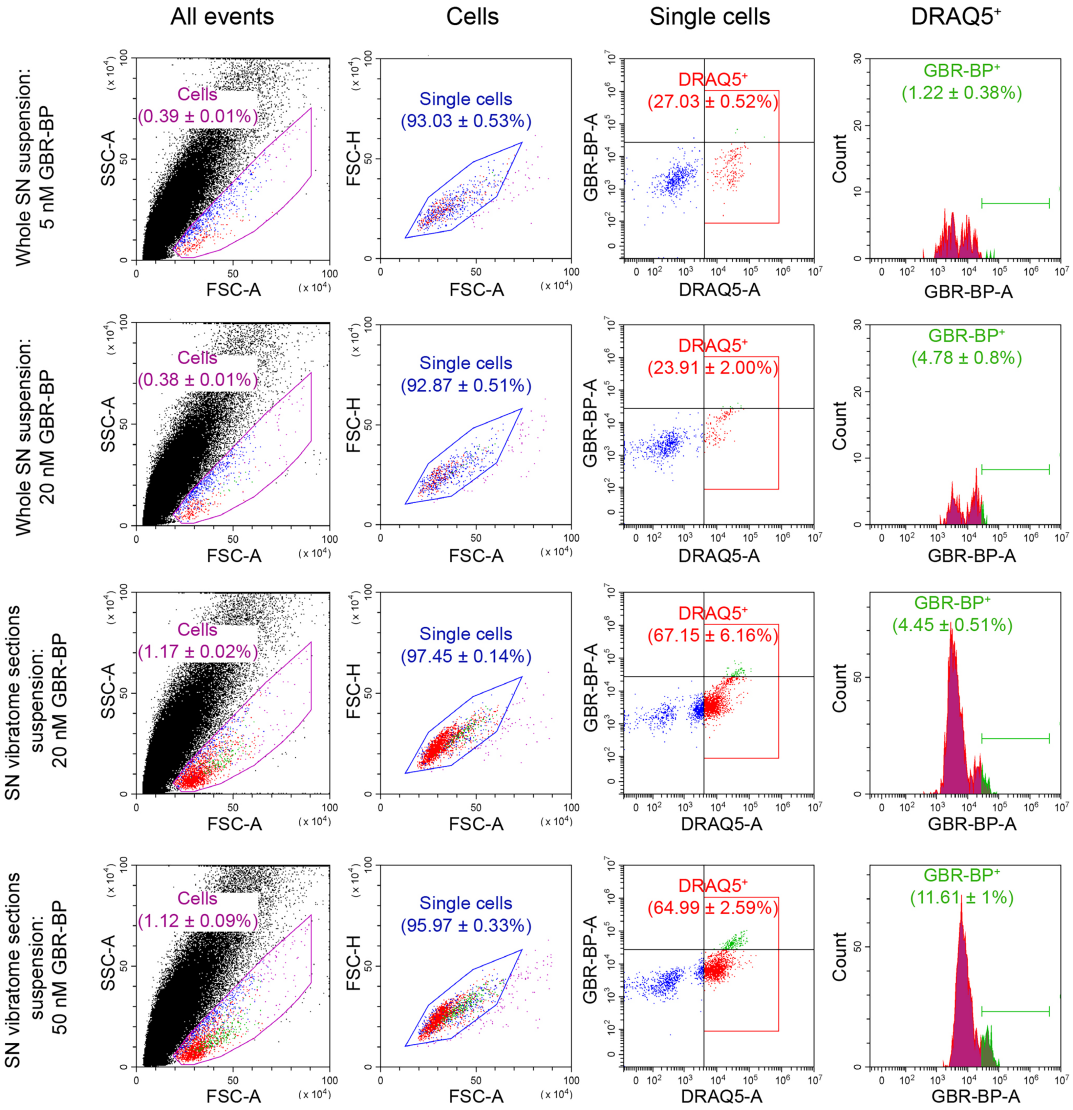
Quantification of substantia nigra (SN) cell suspension by flow cytometry after using various methods for cell dissociation and staining with DRAQ5 and GBR-BP. For each measurement *n* = 3. CTCF, corrected total cell fluorescence; DRAQ5^+^, DRAQ5-stained cells; FSC, forward scatter; GBR-BP^+^, GBR-BP-stained cells; SSC, side scatter.

**Figure 3:**
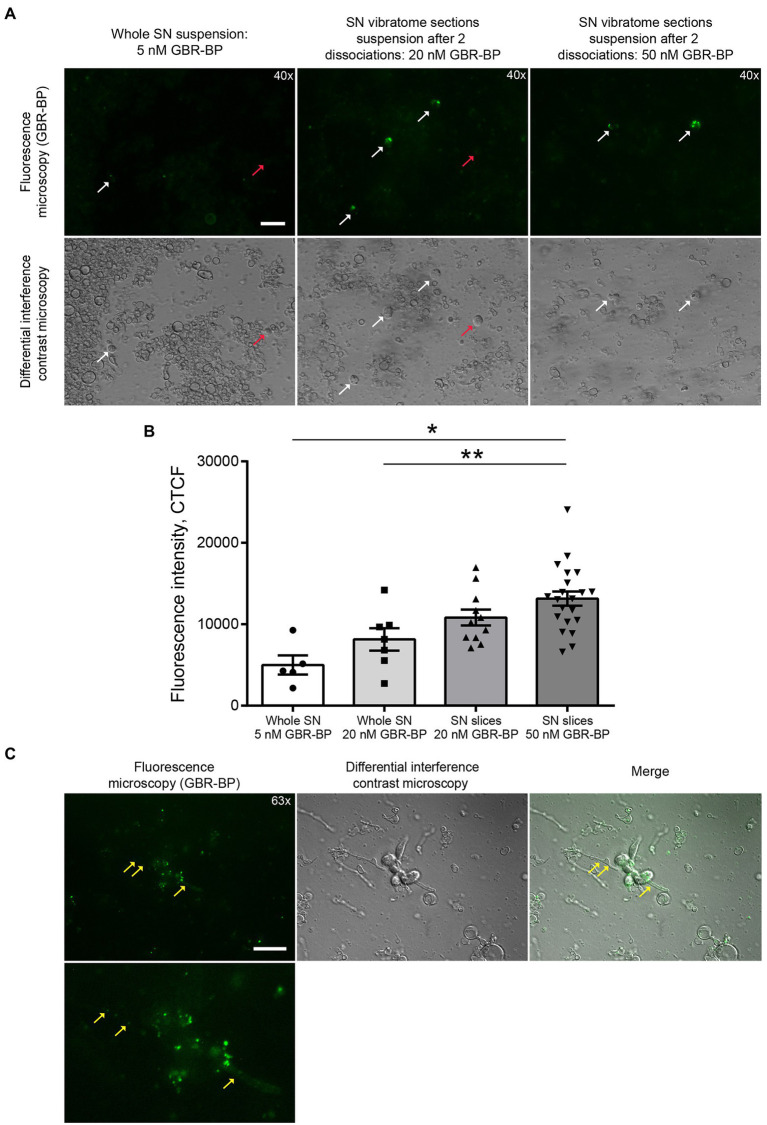
GBR-BP staining of dopaminergic neurons in the substantia nigra (SN). **(A)** Fluorescence microscopy and differential interference contrast microscopy of SN cell suspensions stained with GBR-BP. White arrows, GBR-BP-stained cells; red arrows, GBR-BP-unstained cells. Scale bar, 20 μm. **(B)** Comparison of fluorescence intensity of GBR-BP-stained cells in cell suspensions. **(C)** Internalization of GBR-BP in dopaminergic neuron cell bodies and processes after dissociation of SN vibratome section suspension stained by 20 nM GBR-BP. Yellow arrows, GBR-BP-stained cell processes. Scale bar, 20 μm. Statistics indicate significance by one-way ANOVA. *p* values were corrected for multiple comparison (Tukey) (* *p* < 0.05 compared with the whole SN 5 nM GBR-BP group; ** *p* < 0.05 compared with the whole SN 20 nM GBR-BP group). Data are presented as mean ± SEM. *n* = 10 field of view for each group.

After the sequential incubation of whole SN cell suspension with 10 μM DRAQ5 and 5 nM GBR-BP, only 1.22% of cells stained with DRAQ5 were also stained with GBR-BP (Figure 2). According to microscopy, the fluorescence intensity of GBR-BP-stained cells was 5,085 ± 1,158 according to CTCF (Figures 3A,B). In the staining specificity control, after pre-incubation of the cell suspension with 10 μM GBR 12909, a DAT inhibitor, and subsequent co-incubation with 10 μM GBR 12909 and 5 nM GBR-BP, no stained cells were detected (Supplementary Figure 4). These data are in good agreement with a previous study, which showed that GBR-BP stains DNs due to its binding to DAT and subsequent internalization of the DAT-GBR-BP complex (Blokhin et al., 2022).

From the above data, it follows that when staining the SN cell suspension with 10 μM DRAQ5 and 5 nM GBR-BP, it is impossible to obtain all population of double-stained cells for analysis by flow cytometry. Therefore, the concentration of GBR-BP was increased to 20 nM while maintaining the same concentration of DRAQ5 (Figure 1A). It is important to note that, according to fluorescence microscopy, the cells of the suspension prepared from the whole SN lack processes. This was also the case for dissociated neurons obtained from other areas of the brain (Fanous et al., 2013; Martin et al., 2017; Miyazaki et al., 2020).

After incubation of the whole SN cell suspension with 10 μM DRAQ5 and 20 nM GBR-BP and subsequent flow cytometry, 4.78% of cells stained with DRAQ5 were also stained with GBR-BP (Figure 2). Although more DRAQ5-stained cells appeared to be also stained with GBR-BP, the fluorescent intensity of not all cells with affinity for GBR-BP exceeded the previously set threshold. An increase in the concentration of GBR-BP from 5 nM to 20 nM did not lead to an increase in the fluorescence intensity on the CTCF scale (Figure 3B). In the control, no stained cells were detected after pre-incubation of the cell suspension with GBR 12909 and subsequent co-incubation with GBR 12909 and 20 nM GBR-BP (Supplementary Figure 4).

One of the hypothetical opportunities to increase the population of isolated GBR-BP-stained DNs was to stain not only the cell bodies of DNs, as we did when using a cell suspension of the whole SN (see above), but also the DN cell processes. Indeed, GBR-BP has been shown to stain both the cell bodies and the processes of DNs in a mouse primary culture of mesencephalon and LUHMES cells (Blokhin et al., 2022). To solve this problem, DNs were stained with GBR-BP in SN vibratome sections after papain treatment until cell dissociation. It was first shown that 200 μm thick vibratome sections are well preserved after incubation with papain and subsequent centrifugation. Due to the small number of DNs in the SN compared to all cells isolated from other brain regions (Guez-Barber et al., 2011; Turko et al., 2019), dissociated cells from SN vibratome sections were precipitated by centrifugation at 500 x g in accordance with Eriksen et al. (2009).

Given the weak cell staining with GBR-BP at a concentration of 5 nM in a suspension of the whole SN, we have stained vibratome sections of the SN with GBR-BP at a concentration of 20 nM (Figure 1A). After incubation of SN vibratome sections with 10 μM DRAQ5 and 20 nM GBR-BP and their subsequent dissociation, 4.45% of DRAQ5-stained cells were also stained with GBR-BP (Figure 2). However, it appears that not all cells with affinity for GBR-BP stain with a fluorescence intensity above the threshold. Comparison of staining with 10 μM DRAQ5 and 20 nM GBR-BP of a cell suspension of whole SN and a cell suspension obtained from SN vibratome sections shows that the percentage of cells stained both with DRAQ5 and GBR-BP is the same (4.5%). However, the total number of double-stained cells obtained from the SN vibratome sections was 745% higher. This is due to a 208% increase in the total cell population obtained from the SN vibratome sections and a 181% increase in the DRAQ5-stained cell population compared to a cell suspension obtained from whole SN. Moreover, the analysis of SN vibratome sections cell suspension stained with 10 μM DRAQ5 and 20 nM GBR-BP in a fluorescence microscope at high magnification (63x) confirmed that GBR-BP stains not only DN cell bodies, but also their processes (Figure 3C). GBR-BP-stained cells were not detected in the control after incubating SN vibratome sections first with 10 μM GBR 12909 and then with 10 μM GBR 12909 and 20 nM GBR-BP (Supplementary Figure 4).

Thus, when cell suspension of vibratome sections of SN was stained with 10 μM DRAQ5 and 20 nM GBR-BP, more double-stained cells were detected compared to the same staining of the whole SN cell suspension.

To increase the fluorescence intensity of GBR-BP-stained cells in the suspension of SN vibratome sections, we increased the concentration of GBR-BP to 50 nM in accordance with a previously published paper (Blokhin et al., 2022), while using the same concentration of DRAQ5 (Figure 1A). After incubation SN vibratome sections with 10 μM DRAQ5 and 50 nM GBR-BP, 11.61% of DRAQ5-stained cells were stained with GBR-BP (Figure 2). In this case, the fluorescence intensity of GBR-BP-stained cells was 158% [*F*(3.39) = 7.23, *p* = 0.0006, *p* = 0.0014] and 61.46% (*p* = 0.0174) higher than the fluorescence intensity of GBR-BP-stained cells in whole SN cell suspensions stained with 10 μM DRAQ5 and 5 nM or 20 nM GBR-BP according to CTCF, respectively (Figure 3B). In the control of specificity, no fluorescent cells were detected after the incubation of SN vibratome sections, first with 10 μM GBR 12909, and then with 10 μM GBR 12909 and 50 nM GBR-BP (Supplementary Figure 4).

From a comparison of the protocols for the dissociation and staining of the cell suspension obtained from the whole SN or SN vibratome sections (Figure 2), it follows that the total cell population and DRAQ5-stained cell population are lower when centrifuged at 300× g than at 500× g (0.3867 ± 0.0076% cells vs. 1.143 ± 0.04216% cells, *p* = 0.0022; 28.28 ± 1.138% DRAQ5-stained cells vs. 79.3 ± 3.113% DRAQ5-stained cells, *p* = 0.0022). It is of particular importance that centrifugation of a suspension prepared from pre-stained SN vibratome sections at 500× g minimizes cell loss and simplifies supernatant collection compared to the most widely used method for precipitating dissociated cells at 300× g (St John et al., 1986; Yamasaki et al., 2018).

Thus, pre-staining SN vibratome sections with 10 μM DRAQ5 and 50 nM GBR-BP leads to the maximum fluorescence intensity of subsequently dissociated cells. This makes it possible to isolate the DN population by flow cytometry or cell sorting.

### Viability of cells obtained by dissociation of the whole substantia nigra and vibratome sections of the substantia nigra

In addition to developing an optimal protocol for dissociating SN and staining cells with DRAQ5 and GBR-BP, we compared the viability of the cell suspensions obtained from the whole SN and SN vibratome sections, stained with 10 μM DRAQ5 and 10 μg/ml PI.

**Figure 4:**
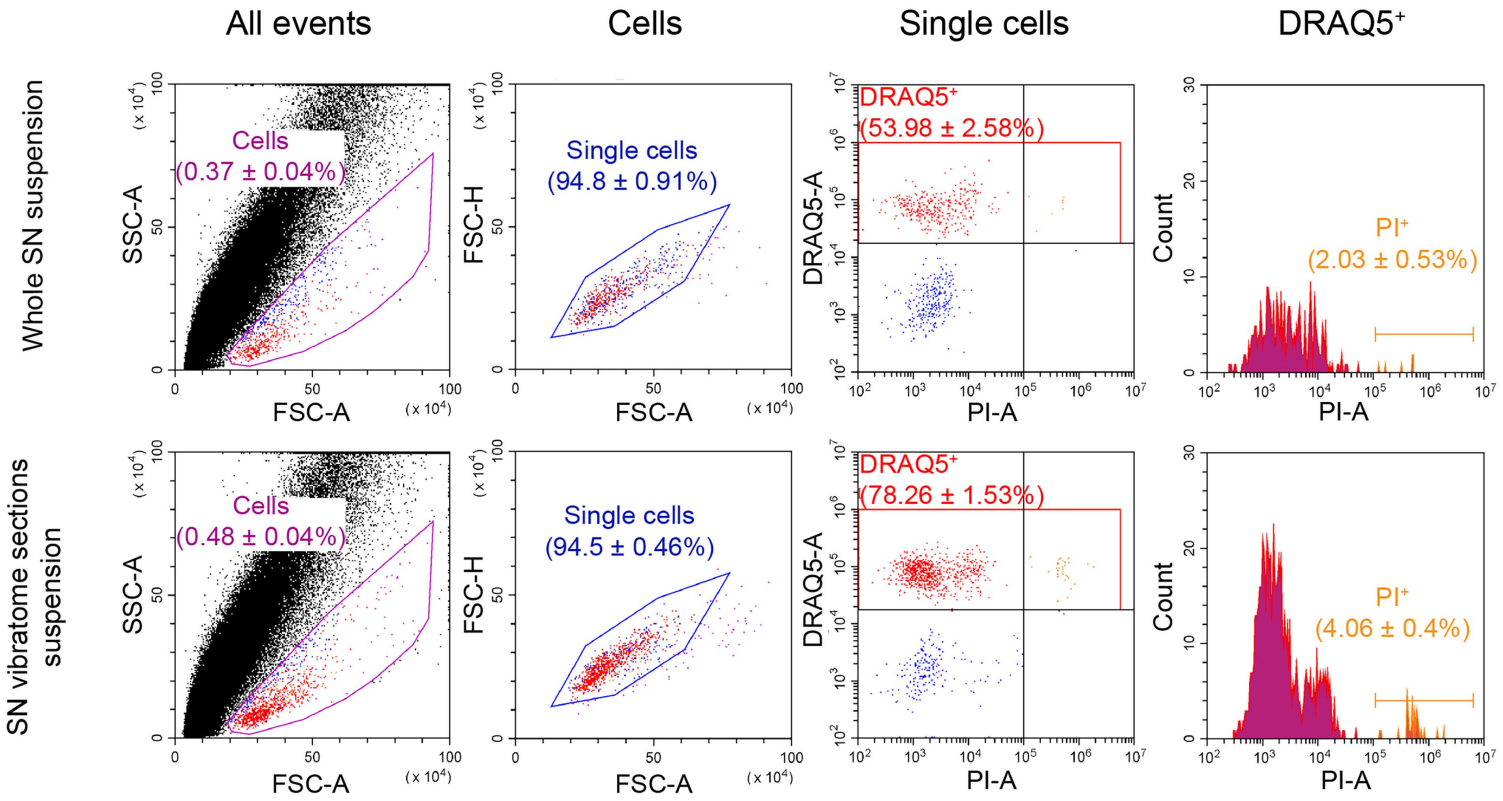
Flow cytometry analysis of cell viability as a relation of dead cells (propidium iodide (PI)-stained) to DRAQ5-stained cells. For each measurement n = 3. FSC, forward scatter; SSC, side scatter.

The proportion of DRAQ5-stained and PI-stained cells obtained from the whole SN suspension was 2.03% of all DRAQ5-stained cells (Figure 4). As for the cell suspension obtained from SN vibratome sections suspension, the proportion of DRAQ5-stained and PI-stained cells were 4.06% of all DRAQ5-stained cells (Figure 4). Thus, the viability of cells obtained by dissociation of the whole SN and SN vibratome sections was not significantly different (*p* = 0.1).

Proceeding from our experience of DNs selection from a cell suspension obtained from the whole SN or SN vibratome sections, we decided to further sort DNs from a suspension of SN vibratome sections prestained with 10 μM DRAQ5 and 50 nM GBR-BP.

### Cell suspension analysis by imaging flow cytometry

**Figure 5:**
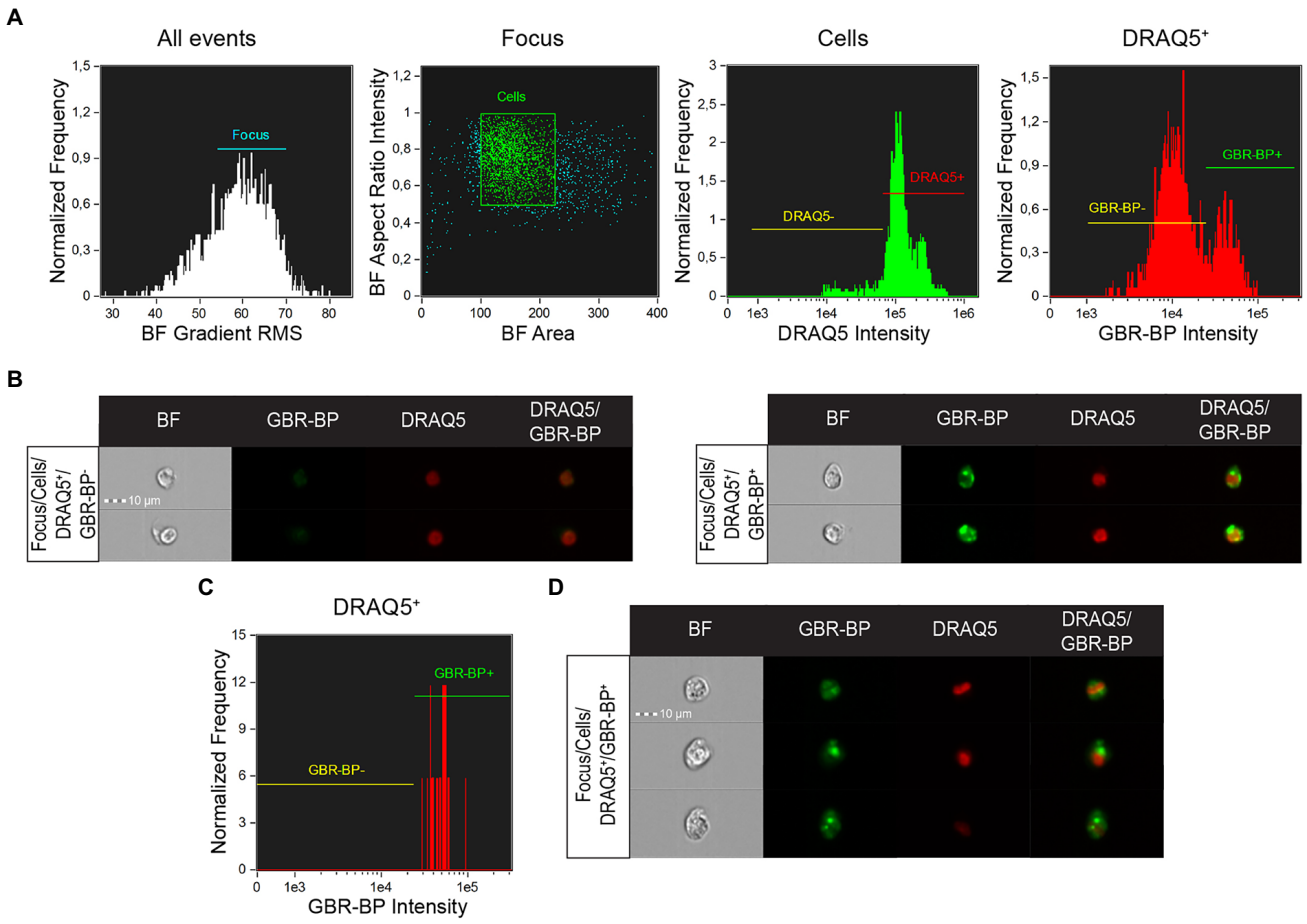
Assessing the sorting efficiency of double-stained (DRAQ5^+^/GBR-BP^+^) cell population by imaging flow cytometry. **(A)** Characteristic of the total cell suspension of substantia nigra (SN) vibratome sections pre-stained with DRAQ5 and GBR-BP (*n* = 1). **(B)** Images of cells from the total cell suspension of SN vibratome sections pre-stained with DRAQ5 and GBR-BP. **(C)** Population of DRAQ5^+^/GBR-BP^+^ cells sorted from the total cell suspension (*n* = 1). **(D)** Images of DRAQ5^+^/GBR-BP^+^ cells sorted from the total cell suspension. BF, bright field; RMS, root mean square.

Cell suspension analysis of SN vibratome sections pre-stained with DRAQ5 and GBR-BP by imaging flow cytometry showed that single focused images in the “Cells” population were predominantly represented by DRAQ5-stained cells (Figure 5A). Among these cells, 2 populations were selected, differing in fluorescence in the green part of the spectrum: GBR-BP-unstained cells (GBR-BP^−^) and GBR-BP-stained (GBR-BP^+^) cells (Figures 5A,B). These data are similar to those obtained when analyzing the suspension on a flow cytometer (Figure 2). Image analysis of sorted cells showed that all DRAQ5-stained cells were also stained with GBR-BP (Figures 5C,D).

Thus, we succeeded in isolating from the SN cell suspension a population of DRAQ5-stained and GBR-BP-stained cells, which are most probably DNs.

### Evidence that cells in the substantia nigra cell suspension sorted by two labels, DRAQ5 and GBR-BP, are dopaminergic neurons

**Figure 6:**
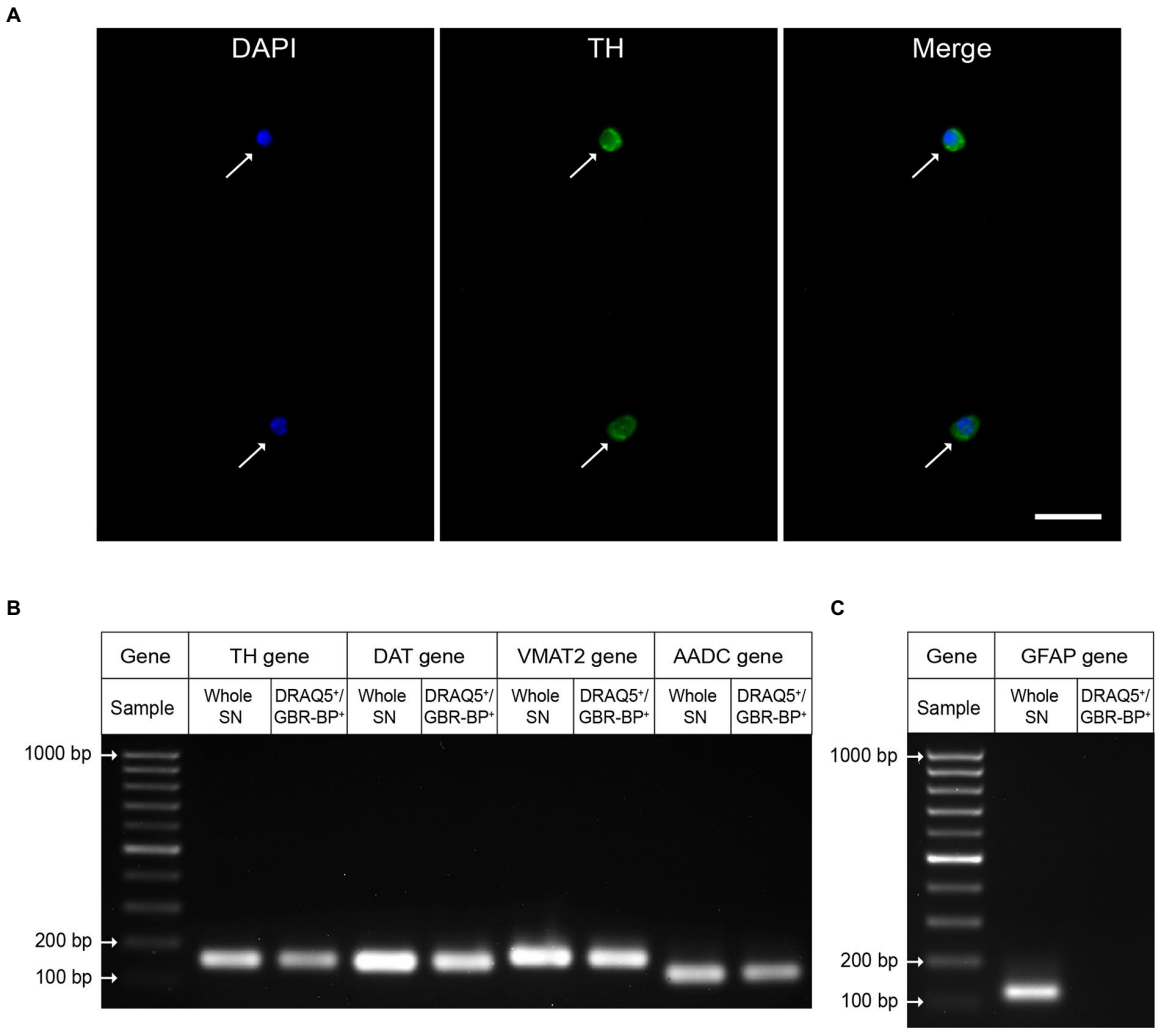
Evidence that the sorted population of double-stained cells (DRAQ5^+^/GBR-BP^+^) are dopaminergic neurons. **(A)** Tyrosine hydroxylase (TH)-immunopositive neurons (green) with 4′,6-diamidino-2-phenylindole (DAPI)-stained nuclei (blue) in the substantia nigra (SN) cell suspension sorted by two fluorescent markers, DRAQ5 and GBR-BP (*n* = 3). Arrows, TH-immunopositive neurons. Scale bar, 20 μm. **(B)** Expression of genes for protein markers of dopaminergic neurons: TH, dopamine transporter (DAT), vesicular monoamine transporter 2 (VMAT2), and aromatic L-amino acid decarboxylase (AADC) in DRAQ5^+^/GBR-BP^+^ cells sorted from a cell suspension of SN vibratome sections, as well as in the SN (homogenate) in intact mice (*n* = 3). **(C)** Gene expression of the glial fibrillary acidic protein (GFAP) (*n* = 3).

To prove that the sorted DRAQ5- and GBR-BP-stained cells of the suspension obtained from SN vibratome sections are DNs, these cells were immunostained for TH, the rate-limiting enzyme of dopamine synthesis. It was shown that 95.46 ± 1.509% of the sorted cells with DAPI-stained nuclei are immunopositive for TH (Figure 6A). Whereas before sorting only 12.64 ± 0.577% of cells were TH-immunopositive (Supplementary Figure 5).

Additional evidence that the sorted cells of dissociated SN vibratome sections are DNs was obtained using qPCR. Indeed, cells stained with DRAQ5 and GBR-BP have been shown to express genes for such DNs marker proteins as TH, DAT, VMAT2, and AADC (Figure 6B). It is important to emphasize that, in contrast to whole SN qPCR results, DRAQ5 and GBR-BP stained cells sorted from SN vibratome slice suspension lack expression of the glial fibrillary acid protein gene, a marker of astrocytes (Figure 6C).

The development of a method for selecting a population of DNs from the SN of intact mice made it possible to move to the last objective of this study: testing changes in gene expression in a sorted DN population in mice in pathology compared with the control.

### Gene expression in a population of dopaminergic neurons selected from substantia nigra in mice in a model of Parkinson’s disease

To test the possibility of assessing the change in gene expression in DNs with a change in the functional state of neurons (2nd objective), we compared the gene expression of specific proteins (TH, DAT, and VMAT2) in the DNs obtained from the SN in mouse model of PD. PD was modeled by four systemic injections of MPTP at a single dose of 12 mg/kg with an interval of 2 h between injections, while control mice received saline.

**Figure 7:**
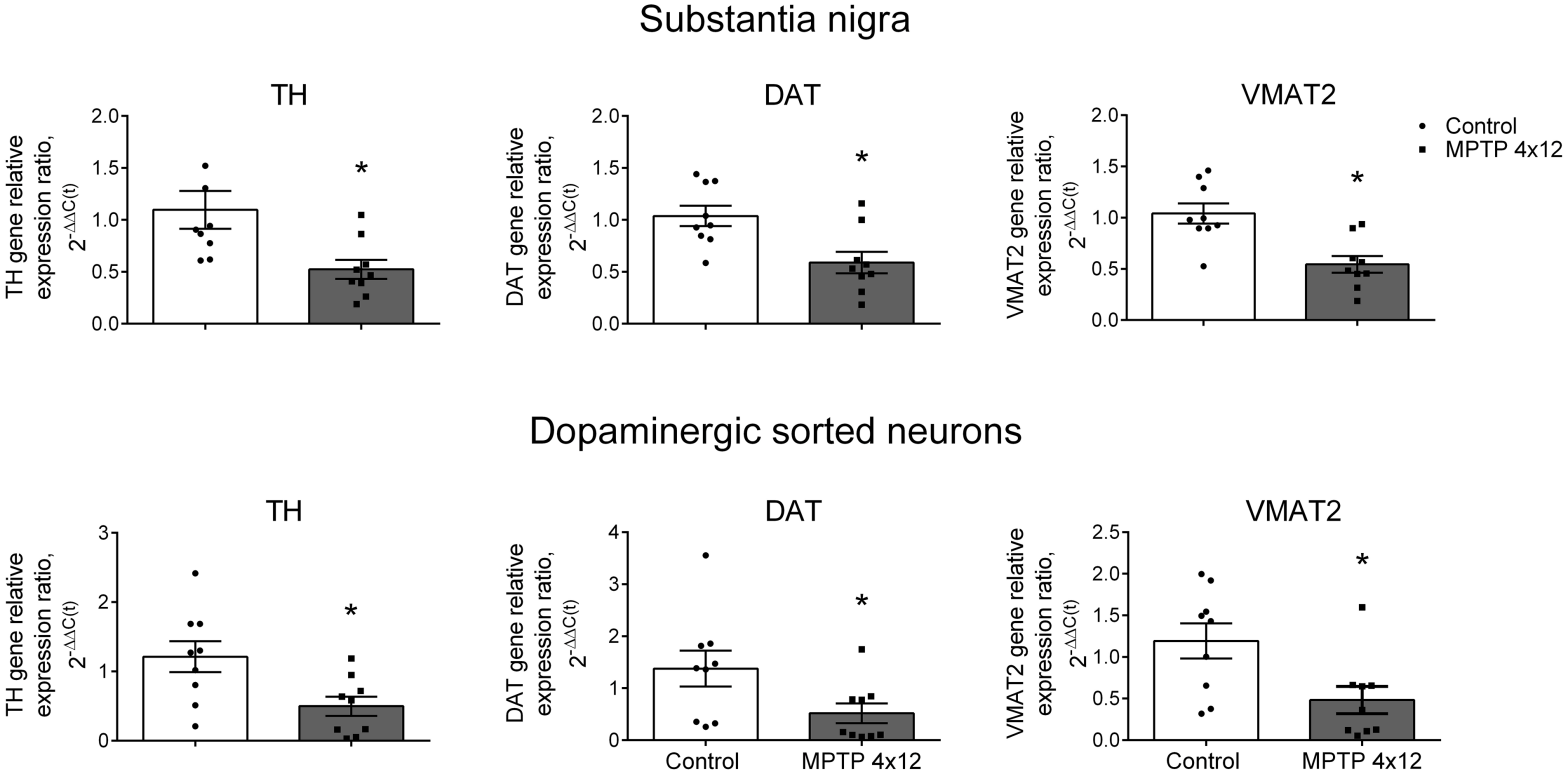
Expression of genes in the whole substantia nigra and sorted dopaminergic neurons in 1-methyl-4-phenyl-1,2,3,6-tetrahydropyridine (MPTP)-treated mice. Expression of the tyrosine hydroxylase (TH) gene, dopamine transporter (DAT) gene, and vesicular monoamine transporter 2 (VMAT2) gene in the whole substantia nigra (homogenate) and sorted dopaminergic neurons in mice 3 weeks after 4-fold administration of MPTP in a single dose of 12 mg/kg, or saline in control. Groups were compared for normality by using the D’Agostino & Pearson test. Statistics indicate significance by the unpaired *t* -test or Mann–Whitney test, depending on the type of distribution (*p < 0.05 compared with the control group). Data are presented as mean ± SEM. *n* = 9 for each group.

First, we assessed the change in TH, DAT and VMAT2 gene expression in the SN homogenate of MPTP-treated and control mice. The cytochrome C1 gene was used as a reference gene, since its expression changes the least during neurodegeneration compared to other genes (Rydbirk et al., 2016). The administration of MPTP to mice led to a downregulation in the expression of the TH gene in the whole SN by 52%, compared with the control taken as 100% (*p* = 0.004; Figure 7). In the same experiment, DAT and VMAT2 gene expression downregulated in the whole SN by 43 and 48%, compared with the control (*p* = 0.0062 and *p* = 0.0013, respectively; Figure 7).

Then, we assessed the change in the TH, DAT and VMAT2 gene expression in DNs sorted from the SN of MPTP-treated and control mice. The expression of the TH gene in DNs of MPTP-treated mice downregulated by 59%, compared with the control (*p* = 0.0156; Figure 7). In the same experiment, a 62% downregulation in DAT gene expression and 60% downregulation in VMAT2 gene expression were found in the sorted DNs of MPTP-treated mice, compared with the control (*p* = 0.0448 and *p* = 0.0244, respectively; Figure 7). The downregulation in expression of the TH, DAT and VMAT2 genes found in sorted DNs in MPTP-treated mice did not differ from that in the whole SN (*p* = 0.99, *p* = 0.51 and *p* = 0.46, respectively; Supplementary Figure 6).

In the same experiment, downregulated expression of the AADC gene, which is not specific for DNs, was observed in the SN homogenate, but not in the sorted DNs (Supplementary Figure 7).

In addition to assessing the changes in the expression of specific genes of DNs, we found that in MPTP-treated mice, the number of sorted DNs was 28.4% less than in the control (*p* = 0.0267; Supplementary Figure 8).

Thus, we have shown unidirectional similar changes in the number of DNs and the expression of specific genes in sorted DNs and in the whole SN in MPTP-treated mice, which opens up broad prospects for studying the expression of other genes of interest in sorted DNs in normal and pathological animals.

## Discussion

The need to obtain a pure population of living DNs from the SN first appeared 30 years ago when trying to implant these neurons in the striatum of PD patients (Björklund and Lindvall, 2017). Recently, some progress has been made in relevant experimental studies by obtaining a fraction of DNs from the SN of transgenic mouse embryos with the GFP reporter under the control of the TH gene promoter (Yoshizaki et al., 2004; Donaldson et al., 2005; Bye et al., 2015). However, this is a very expensive method due to the high cost of the transgenic mice themselves and their maintenance in a GLP vivarium. With such an approach, only a small amount of material (SN) can be obtained to solve only local research problems. Therefore, in this study, to develop a method for obtaining isolated DNs, we have planned to use much cheaper wild-type mice, which can be kept in a large number under the usual vivarium conditions. This will make it possible to obtain a large number of DNs from the SN, which can be used to solve not only research but also applied problems, such as testing new technologies and drugs. Moreover, this method can be easily adapted to isolate DNs from both the SN and other dopaminergic centers of the brain in any animal species, including non-human primates.

The method we have developed for obtaining the DN fraction opens up new opportunities for studying *ex vivo* those aspects of the functioning of DNs that cannot be studied using current approaches. First of all, this concerns the expression of genes that are not specific for DNs, but play an important role in their functioning. These include genes encoded proteins that are involved: in the synthesis of dopamine – AADC, in the degradation of dopamine – monoamine oxidases, in dopamine neurotransmission – proteins of the vesicular cycle, in the nervous and neuroendocrine regulation – receptors for classical neurotransmitters, neuropeptides, hormones of peripheral endocrine glands. Selection the DN fraction in animals using experimental models of pathology, for example, PD, will also allow to evaluate the gene expression of proteins involved in neurodegeneration and neuroplasticity, the ubiquitin-proteasome system, autophagy, apoptosis, growth factors, heat shock proteins (McNaught et al., 2001; Olanow and McNaught, 2006; Kampinga and Bergink, 2016; Bekker et al., 2021; Wie et al., 2021).

At the first stage of isolation of the DN population, the SN was dissociated approximately in the same way as a cell suspension of other parts of the brain was previously obtained (Xu et al., 2012; Miyazaki et al., 2020). It should be emphasized that the know-how of our method lies in the specific staining of living DNs with a dopamine uptake inhibitor GBR 12909 coupled to the fluorophore, BODYPY. The manufactures of GBR-BP have shown that it specifically binds to DAT and, together with DAT, is internalized and accumulated in DNs (Blokhin et al., 2022). Moreover, it has been shown that GBR-BP is not cytotoxic, and a high intensity of DN staining is maintained for a long time (Blokhin et al., 2022). The second important step in the development of the DN selection method was the staining of GBR-BP not in SN cell suspension, but in SN vibratome sections treated with papain. Such a technical approach made it possible to preserve and stain, in addition to the cell bodies of DNs, their processes. This resulted in a significant increase in GBR-BP signal intensity from stained neurons. Since it was planned to use the DN fraction of SN primarily to assess gene expression, it was necessary to select only DN cell bodies, which contain nuclei. For this, a nuclear dye, DRAQ5, was used in addition to GBR-BP.

The general success in developing a method for obtaining the DN fraction is evidenced by: (i) immunopositivity for TH of 95% of sorted cells; (ii) expression in sorted cells of genes not only for specific proteins of the dopaminergic phenotype, TH, DAT, and VMAT2, but also for nonspecific proteins, such as AADC; (iii) the absence of glial fibrillary acid protein gene expression, which is characteristic of glial cells – astrocytes. It is noteworthy that the use of this method can be extended to serotonergic and noradrenergic neurons of the brainstem, since they also specifically stain with GBR-BP, but at a higher concentration (Blokhin et al., 2022).

When meeting the last objective of this study, we had to check whether the changes in gene expression registered in the sorted DNs and in the SN homogenate would be similar in the pathology of the nigrostriatal dopaminergic system. To do this, we used an acute MPTP model of the early clinical stage of PD, which was previously developed in mice by four subcutaneous injection of MPTP at a single dose of 12 mg/kg with an interval of 2 h between injections (Ugrumov et al., 2011). In accordance with the data obtained, the same downregulation of the expression of TH, DAT, and VMAT2 genes was found in the isolated DNs and in the SN homogenate in MPTP-treated mice, compared with the control mice.

In sum, we have developed a new method for obtaining a pure fraction of living DNs from whole SN. To isolate DNs from the SN cell suspension, they were stained with two fluorescent dyes, DRAQ5 (cell nucleus stain) and GBR-BP (a specific stain for living DNs). This method provides a unique opportunity to evaluate the gene expression of functionally important proteins in DNs, which are also expressed in other cells of the SN (proteins of the ubiquitin-proteasome system, proteins of the vesicular cycle, etc.). The efficiency of this method was confirmed by the discovery of the same downregulation in the expression of genes of proteins specific for DNs (TH, DAT, and VMAT2) in the isolated DNs and in the homogenate of the SN in the MPTP mouse model of PD, compared with the control. Based on the paradigm of translational medicine, the developed method will serve to identify new molecular mechanisms of the pathogenesis of PD, which can be considered as targets for the development of new methods for diagnosing and treating PD.

## Data availability statement

The raw data supporting the conclusions of this article will be made available by the authors, without undue reservation.

## Ethics statement

The animal study was reviewed and approved by Animal Care and Use Committee of the Koltzov Institute of Developmental Biology RAS.

## Author contributions

MU: supervision, funding acquisition, project administration, and manuscript preparation with the participation of DT and VB. MU, DT, and VB: conceptualization and methodology. DT, AK, and VB: cell suspension preparation. VU: cell sorting. DT: microscopy, image analysis, and preparation of illustrations. All authors contributed to the article and approved the submitted version.

## Funding

This research was funded by the Ministry of Science and Higher Education of the Russian Federation (grant agreement.075-15-2020-795, state contract.13.1902.21.0027 of 29.09.2020, unique project ID: RF-190220 × 0027).

## Conflict of interest

The authors declare that the research was conducted in the absence of any commercial or financial relationships that could be construed as a potential conflict of interest.

## Publisher’s note

All claims expressed in this article are solely those of the authors and do not necessarily represent those of their affiliated organizations, or those of the publisher, the editors and the reviewers. Any product that may be evaluated in this article, or claim that may be made by its manufacturer, is not guaranteed or endorsed by the publisher.

## Footnotes

^1^ https://imagej.net/software/fiji/downloads

## Supplementary material

SUPPLEMENTARY FIGURE 1 Analysis of DRAQ5 and GBR-BP unstained substantia nigra cell suspension by flow cytometry. FSC, forward scatter; SSC, side scatter.

SUPPLEMENTARY FIGURE 2 Analysis of DRAQ5 and propidium iodide (PI) unstained substantia nigra cell suspension (Unstained control) and single stain controls (DRAQ5 and PI) by flow cytometry. FSC, forward scatter; SSC, side scatter.

SUPPLEMENTARY FIGURE 3 Analysis of DRAQ5 and GBR-BP unstained substantia nigra cell suspension by imaging flow cytometry. BF, bright field; RMS, root mean square.

SUPPLEMENTARY FIGURE 4 Testing the specificity of GBR-BP staining of cell suspensions of whole substantia nigra (SN) and cell suspensions of SN vibratome sections by co-incubation of cells with GBR-BP and GBR-12909. Arrows, cells unstained with GBR-BP in the presence of GBR-12909. Scale bar, 50 µm.

SUPPLEMENTARY FIGURE 5 Immunocytochemistry of substantia nigra (SN) cell suspension before sorting. Tyrosine hydroxylase (TH)-immunopositive neurons (green) with 4’,6-diamidino-2-phenylindole (DAPI)-stained nuclei (blue) in the SN cell suspension obtained from SN vibratome sections. Arrows, TH-immunopositive neurons. Scale bar, 20 µm.

SUPPLEMENTARY FIGURE 6 Tyrosine hydroxylase (TH), dopamine transporter (DAT), and vesicular monoamine transporter 2 (VMAT2) gene expression normalized to control in the whole SN (homogenate) and sorted dopaminergic neurons in mice two weeks after the administration of 1-methyl-4-phenyl-1,2,3,6-tetrahydropyridine (MPTP) four times at a single dose of 12 mg/kg or saline in the control. Groups were compared for normality by using the D’Agostino & Pearson test. Statistics indicate significance by the unpaired t-test or Mann-Whitney test, depending on the type of distribution. Data are presented as mean ± SEM. n = 9 for each group.

SUPPLEMENTARY FIGURE 7 Expression of the aromatic L-amino acid decarboxylase (AADC) gene in the whole substantia nigra (homogenate) and sorted dopaminergic neurons in mice two weeks after 4-fold administration of 1-methyl-4-phenyl-1,2,3,6-tetrahydropyridine (MPTP) in a single dose of 12 mg/kg, or saline in control. Groups were compared for normality by using the D’Agostino & Pearson test. Statistics indicate significance by the unpaired t-test (* P < 0.05 compared with the control group). Data are presented as mean ± SEM. n = 9 for each group.

SUPPLEMENTARY FIGURE 8 The number of sorted dopaminergic neurons in mice two weeks after 4-fold administration of 1-methyl-4-phenyl-1,2,3,6-tetrahydropyridine (MPTP) in a single dose of 12 mg/kg, or saline in control. Groups were compared for normality by using the D’Agostino & Pearson test. Statistics indicate significance by the unpaired t-test (* P < 0.05 compared with the control group). Data are presented as mean ± SEM. n = 9 for each group.

## Abbreviations

AADC: aromatic L-amino acid decarboxylase
CTCF: corrected total cell fluorescence
DAPI: 4’,6-diamidino-2-phenylindole
DAT: dopamine transporter
DN(s): dopaminergic neuron(s)
FACS: fluorescence-activated cell sorting
GBR-BP: GBR-BODIPY
HBSS: Hank’s balanced salt solution
MPTP: 1-methyl-4-phenyl-1,2,3,6-tetrahydropyridine hydrochloride; PD, Parkinson’s disease
PI: propidium iodide
qPCR: quantitative polymerase chain reaction
SN: substantia nigra
TH: tyrosine hydroxylase
VMAT2: vesicular monoamine transporter 2
