# Supplementary figures and images for: Isolation of living dopaminergic neurons labeled with a fluorescent ligand of the dopamine transporter from mouse substantia nigra as a new tool for basic and applied research
Source: Front Mol Neurosci. 2022 Dec 9;15:1020070. doi: 10.3389/fnmol.2022.1020070 (PMC9780273; doi:10.3389/fnmol.2022.1020070)

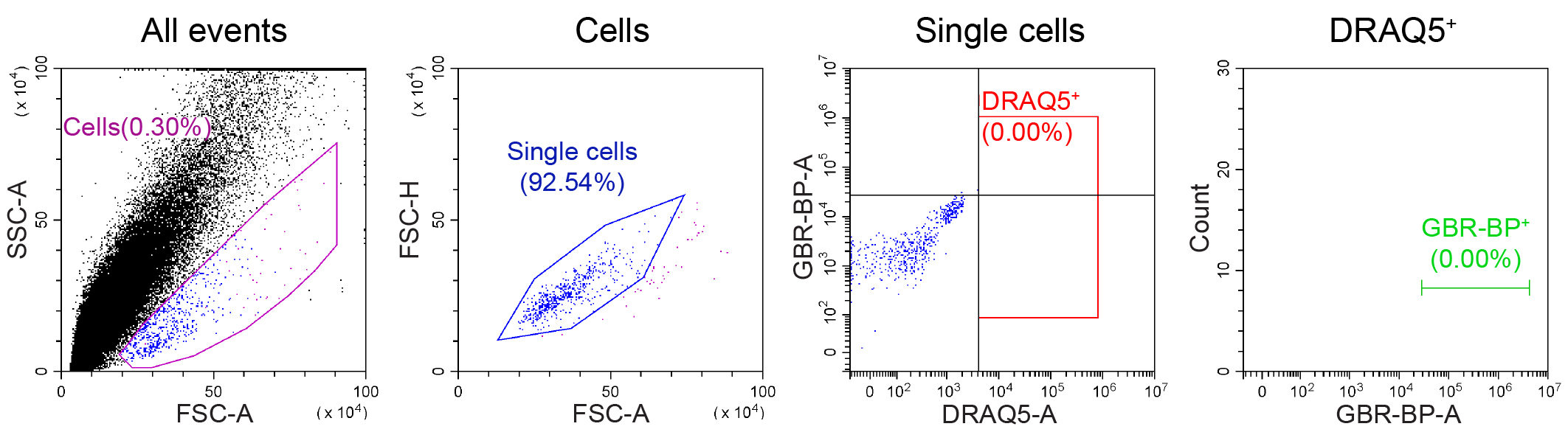

Supplement: SUPPLEMENTARY FIGURE 1 — Analysis of DRAQ5 and GBR-BP unstained substantia nigra cell suspension by flow cytometry. FSC, forward scatter; SSC, side scatter. [file Image_1.JPEG]

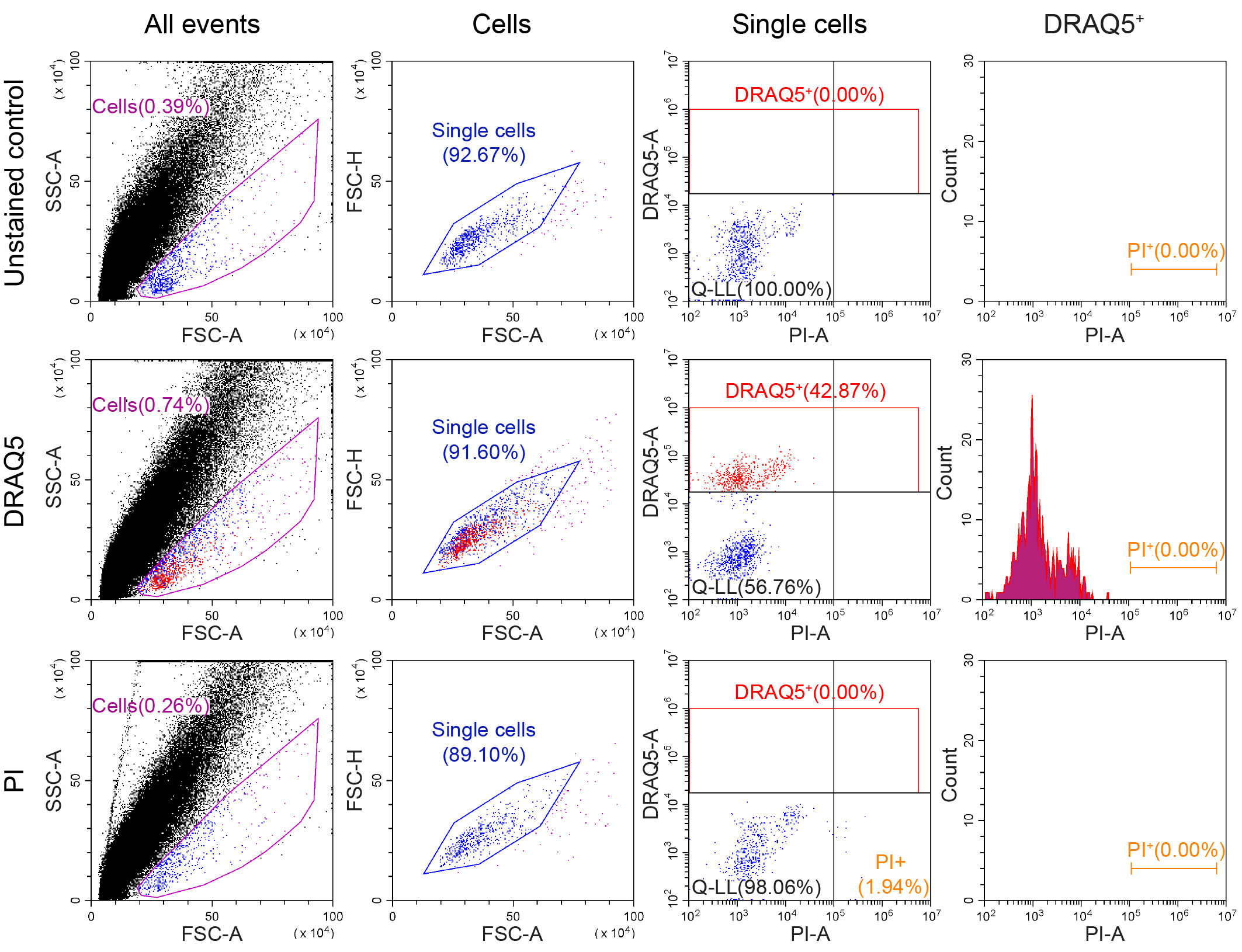

Supplement: SUPPLEMENTARY FIGURE 2 — Analysis of DRAQ5 and propidium iodide (PI) unstained substantia nigra cell suspension (Unstained control) and single stain controls (DRAQ5 and PI) by flow cytometry. FSC, forward scatter; SSC, side scatter. [file Image_2.JPEG]

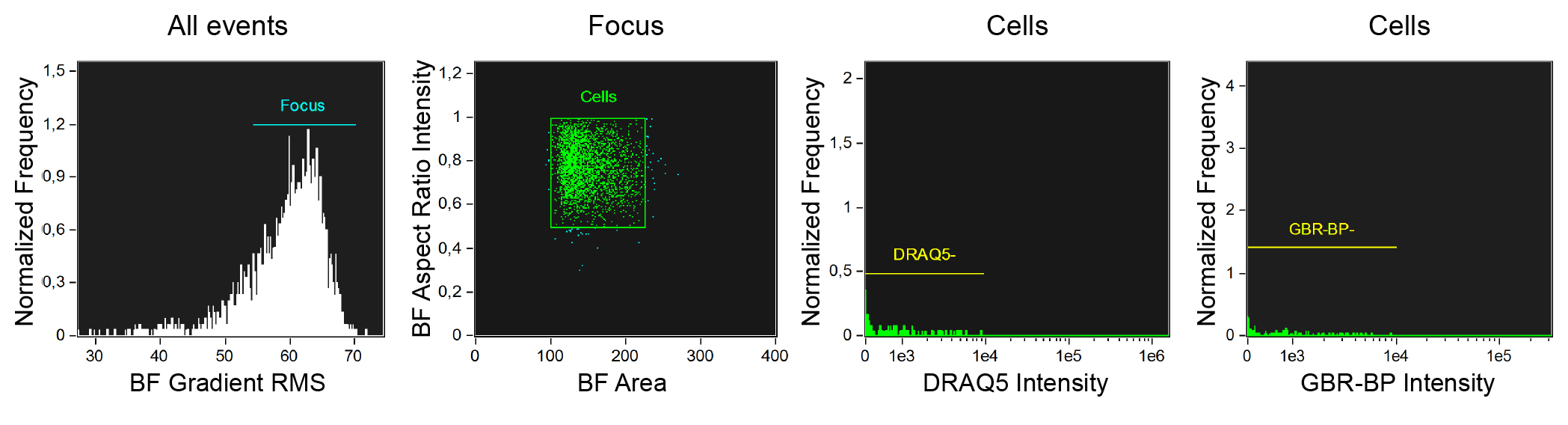

Supplement: SUPPLEMENTARY FIGURE 3 — Analysis of DRAQ5 and GBR-BP unstained substantia nigra cell suspension by imaging flow cytometry. BF, bright field; RMS, root mean square. [file Image_3.JPEG]

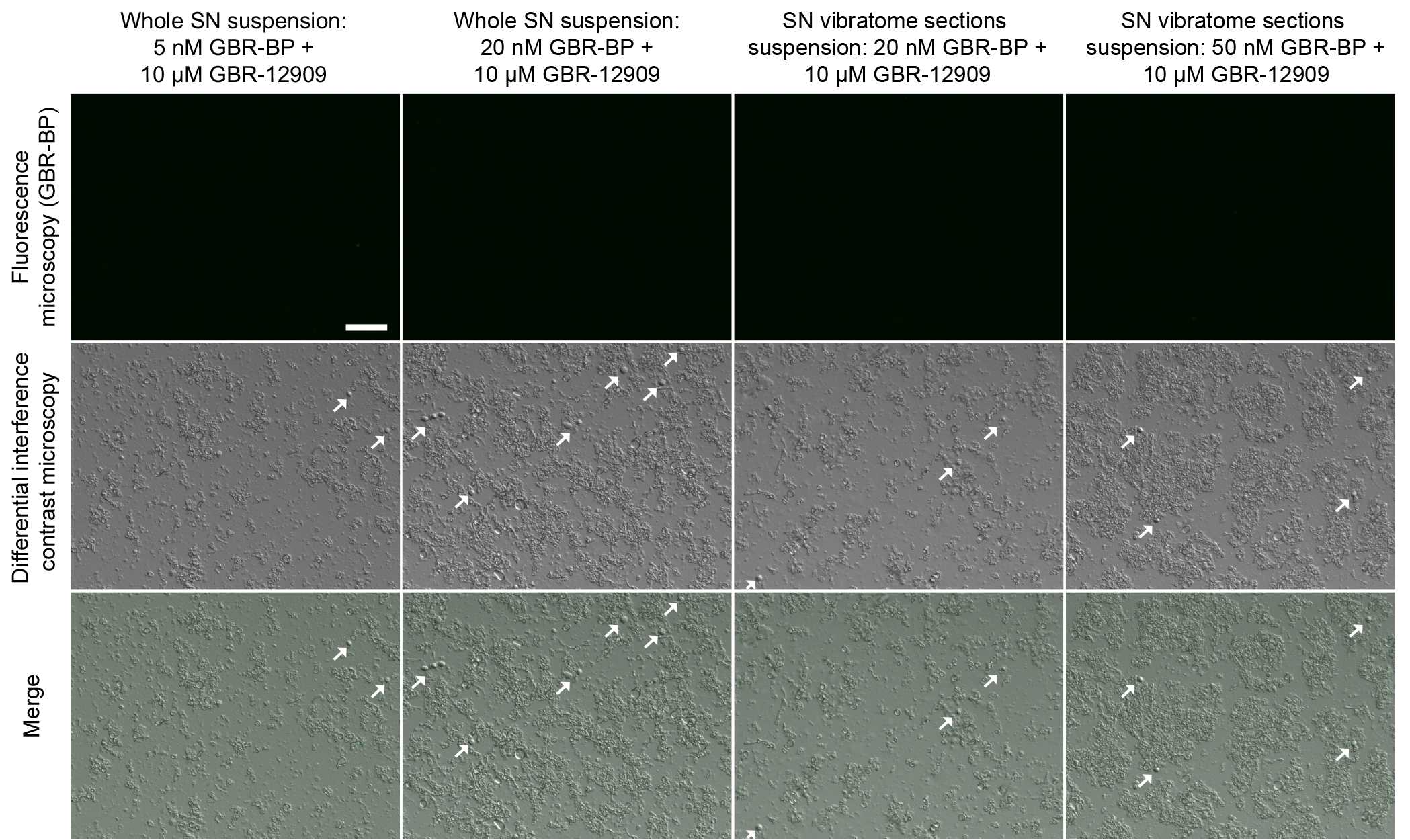

Supplement: SUPPLEMENTARY FIGURE 4 — Testing the specificity of GBR-BP staining of cell suspensions of whole substantia nigra (SN) and cell suspensions of SN vibratome sections by co-incubation of cells with GBR-BP and GBR-12909. Arrows, cells unstained with GBR-BP in the presence of GBR-12909. Scale bar, 50 µm. [file Image_4.JPEG]

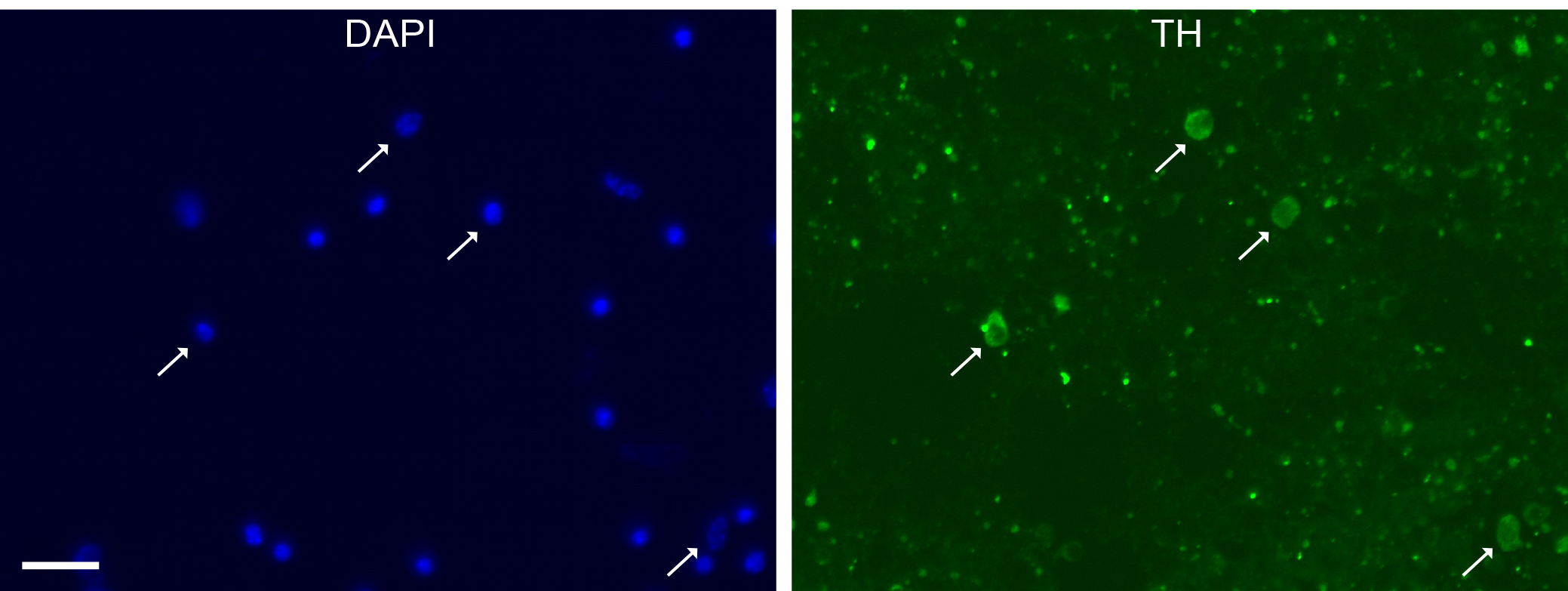

Supplement: SUPPLEMENTARY FIGURE 5 — Immunocytochemistry of substantia nigra (SN) cell suspension before sorting. Tyrosine hydroxylase (TH)-immunopositive neurons (green) with 4’,6-diamidino-2-phenylindole (DAPI)-stained nuclei (blue) in the SN cell suspension obtained from SN vibratome sections. Arrows, TH-immunopositive neurons. Scale bar, 20 µm. [file Image_5.JPEG]

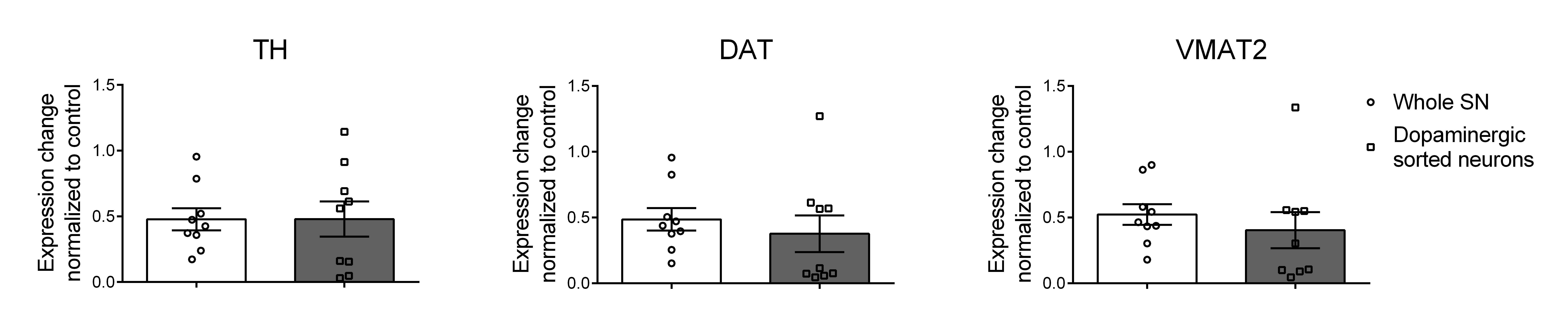

Supplement: SUPPLEMENTARY FIGURE 6 — Tyrosine hydroxylase (TH), dopamine transporter (DAT), and vesicular monoamine transporter 2 (VMAT2) gene expression normalized to control in the whole SN (homogenate) and sorted dopaminergic neurons in mice two weeks after the administration of 1-methyl-4-phenyl-1,2,3,6-tetrahydropyridine (MPTP) four times at a single dose of 12 mg/kg or saline in the control. Groups were compared for normality by using the D’Agostino & Pearson test. Statistics indicate significance by the unpaired t-test or Mann-Whitney test, depending on the type of distribution. Data are presented as mean ± SEM. n = 9 for each group. [file Image_6.JPEG]

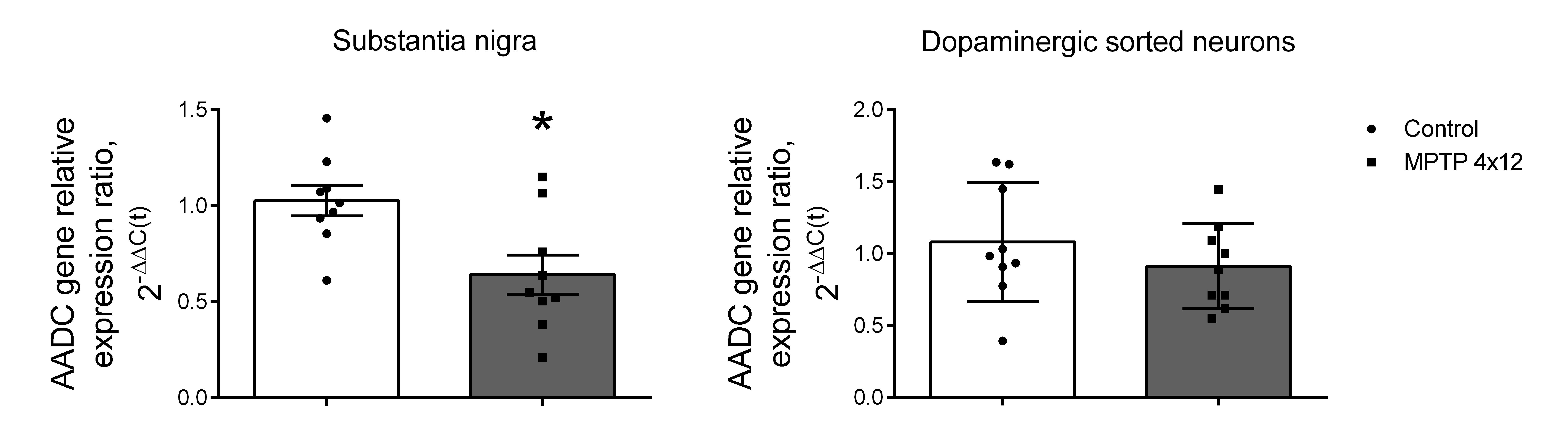

Supplement: SUPPLEMENTARY FIGURE 7 — Expression of the aromatic L-amino acid decarboxylase (AADC) gene in the whole substantia nigra (homogenate) and sorted dopaminergic neurons in mice two weeks after 4-fold administration of 1-methyl-4-phenyl-1,2,3,6-tetrahydropyridine (MPTP) in a single dose of 12 mg/kg, or saline in control. Groups were compared for normality by using the D’Agostino & Pearson test. Statistics indicate significance by the unpaired t-test (* P < 0.05 compared with the control group). Data are presented as mean ± SEM. n = 9 for each group. [file Image_7.JPEG]

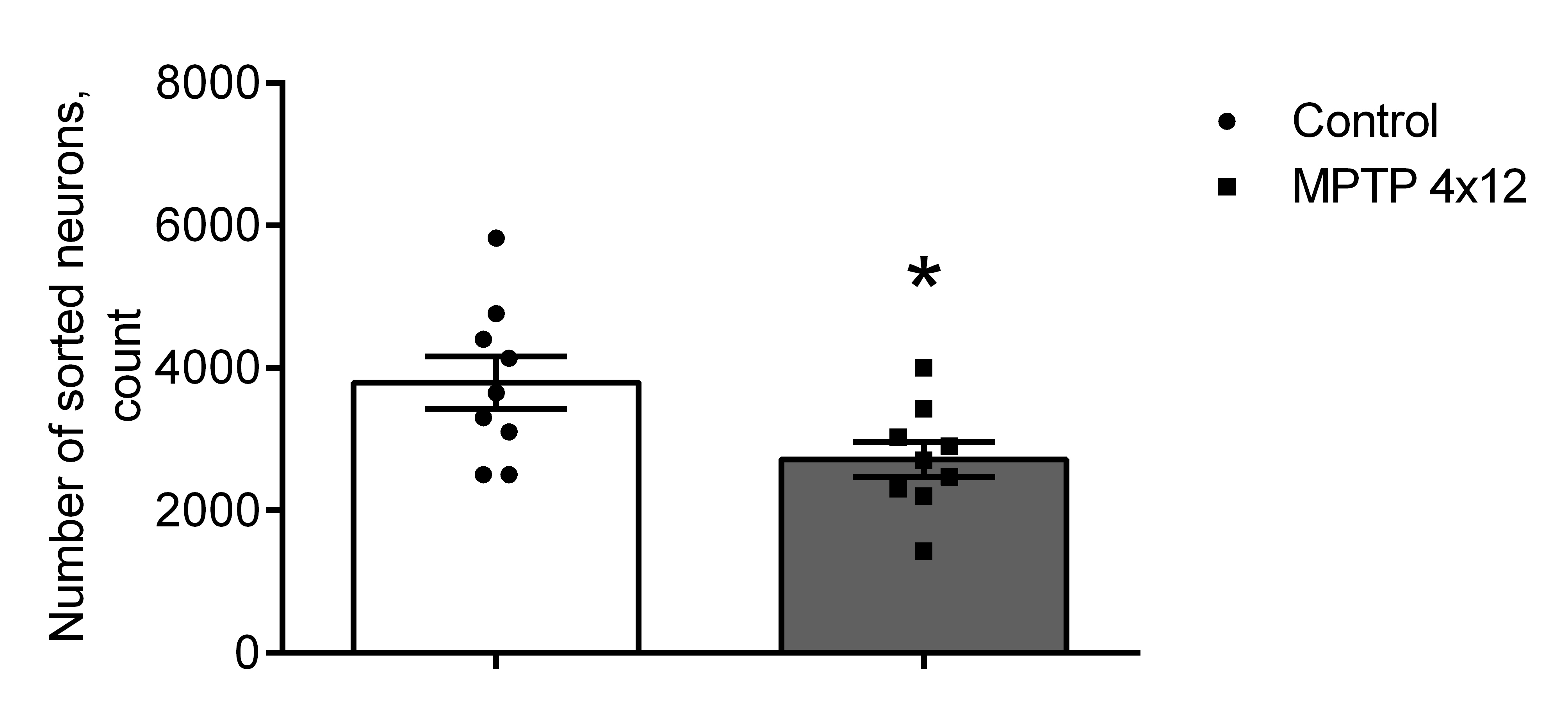

Supplement: SUPPLEMENTARY FIGURE 8 — The number of sorted dopaminergic neurons in mice two weeks after 4-fold administration of 1-methyl-4-phenyl-1,2,3,6-tetrahydropyridine (MPTP) in a single dose of 12 mg/kg, or saline in control. Groups were compared for normality by using the D’Agostino & Pearson test. Statistics indicate significance by the unpaired t-test (* P < 0.05 compared with the control group). Data are presented as mean ± SEM. n = 9 for each group. [file Image_8.JPEG]
